# The Developmental Process of Peer Support Networks: The Role of Friendship

**DOI:** 10.3389/fpsyg.2021.615148

**Published:** 2021-01-28

**Authors:** Lingfei Wang, Lichan Liang, Zhengguang Liu, Keman Yuan, Jiawen Ju, Yufang Bian

**Affiliations:** ^1^Collaborative Innovation Center of Assessment for Basic Education Quality, Beijing Normal University, Beijing, China; ^2^Child and Family Education Research Center, Beijing Normal University, Beijing, China; ^3^Institute of Mental Health and Education, Beijing Normal University, Beijing, China; ^4^Department of Psychology and Behavioral Science, Zhejiang University, Hangzhou, China

**Keywords:** peer support, friendship, peer support network, social network analysis, adolescents

## Abstract

This study investigated the characteristics and development of peer support networks in an effort to unravel the role of friendship in this developmental process. The relationships between friendship networks and peer support networks were explored, and the influence of dyadic and triadic friendships on the development of peer support relationships was examined. Two waves of data were collected among a sample of adolescents in six Chinese junior high schools (*n* = 913 students from 28 classrooms; mean age = 14.13 years; 50.49% boys), and classroom friendship networks and peer support networks were analyzed. The results showed that peer support networks were sparse, hierarchical, and sex-segregated. Furthermore, peer support networks and friendship networks partially overlapped. Friends tended to have similar support-seeking and support-providing ties. Longitudinal multiplex social network analysis revealed that peer support networks changed moderately over time, and friendships played various roles in the development of peer support networks. Dyadic friendships improved the formation of peer support ties. A mutual friend improved the formation of support relationships between two students when the mutual friend chose the two students as friends, but a mutual friend also hindered or had no effects on the formation of support relationships in other cases. The implications for educators to improve peer support networks are presented, and directions for future research are discussed.

## Introduction

Adolescents are usually confronted with growing stress and challenges because they are at physiological and psychological turning points in their lives. Social support is beneficial for them to handle these challenges. Parents, teachers, and peers usually provide different types of social support. Peers mainly offer emotional and informational support (Hombrados-Mendieta et al., 2012; Wentzel et al., 2016). Researchers have found that adolescents stay late at school and go out with peers often (Larson et al., 1996; Tarrant, 2002), and they usually seek support from peers to solve problems (Bokhorst et al., 2010). Researchers have also found that social support from peers increases while support from parents and teachers decreases across the entire period of adolescence (Bokhorst et al., 2010; Hombrados-Mendieta et al., 2012). Thus, peers are an important source of social support for adolescents.

Support from peers is called peer support, which can be defined as emotional or educational support and a “listening ear” for others in a peer group (Cowie, 2019). Adolescents who receive more peer support often report fewer behavioral problems (McElhaney et al., 2006; Holt and Espelage, 2007), have better academic outcomes (Elias and Haynes, 2008; Song et al., 2015), and experience more life satisfaction (Danielsen et al., 2009) and less psychological distress, such as anxiety and depressive symptoms (Desjardins and Leadbeater, 2011; Coyle et al., 2017). Overall, peer support plays an important role in the development of adolescents.

Adolescents usually play and study in peer groups and receive support from group members (Kindermann and Gest, 2009). According to ecological systems theory (Bronfenbrenner and Morris, 2007), the peer group is a microsystem in which individuals act and communicate with others, and the interplay between individuals, other members, and the environment influences the development of individuals and the environment (e.g., the development of individuals’ peer support). For example, students tend to receive more support when they are in a supportive school climate (Shim et al., 2013; Connolly and Corcoran, 2016), and peers affect others in peer support behavior to improve the supportive climate (Shin, 2018). Therefore, studies of peer support in this microsystem should include characteristics of individuals, relationships among peers, and features of the group.

Peer support networks provide a way to investigate peer support ecologically and systematically and have some unique attributes in peer support research. A peer support network comprises members of a peer group and support relationships among members (Lee et al., 2016; van Rijsewijk et al., 2020). In the network, people seek, receive, and provide peer support through these relationships (Barbee and Cunningham, 1995). Although few studies have investigated peer support networks, the extant studies of social support networks and social network analysis have indicated that peer support networks may have several unique qualities. First, researchers can explore interactions between support seekers and support providers and the role of dyadic relationships on peer support networks. For example, a previous study explored the formation of the received support relationship between two students and the role of friendship and sex among students in peer support networks (van Rijsewijk et al., 2020). This implies that seekers, providers, and their relationships can be studied as a system instead of exploring support relationships only from the seeker’s or provider’s perspective. Second, researchers can investigate the role of environmental factors in the development of peer support relationships. For example, earlier studies explored the ways in which other persons in a network influence the formation of friendship and bullying relationships between two members (Rambaran et al., 2019) and the ways in which characteristics of classrooms influence the development of friendships among students (Laninga-Wijnen et al., 2017). In summary, research on peer support networks can elucidate support relationships in a group, and support relationships can be explored ecologically and systematically. Additionally, because relationships between two members in a network are usually called a “tie” in social network analysis, this term is used below.

## The Developmental Process of Peer Support Networks

Peer support relationships in networks are continually created, dissolved, and maintained over time (Zander et al., 2019), implying that peer support networks are always changing and developing. By exploring the developmental process of peer support networks, researchers can better understand the developmental characteristics of these networks and identify key problems in the development of such networks. Researchers can also search for ways to improve peer support networks and promote mental and physical health among their members.

Several perspectives could be used to explore the developmental process of peer support networks. First, drawing on research on peer networks, researchers could explore the overall condition of support relationships in a group by network indices and investigated the development of network indices, such as the density and number of supporters. Studies have found that the density of peer networks decreases and reciprocity increases from the first semester to the second semester in both boys and girls in the fifth grade, and the number of mutual ties and friends (i.e., opting in or opting out) in peer networks is not the same in different school grades (Urberg et al., 1995; Gest et al., 2007). These studies have shown that this method can reveal the overall developmental trend of peer networks, implying that we can use this method to explore peer support networks. Second, another research approach for studying the development of peer support networks is to explore the developmental process of local structures. These local structures reveal local regularities in a network, namely the regularities of relationships among several members in this network, such as the activity of a member and transitive relationships among several members (Lusher and Robins, 2013). Considering that networks consist of local structures (e.g., dyadic relationships), researchers could explore the developmental process of these local structures to understand the developmental process of networks (Lusher and Robins, 2013).

Researchers can then improve peer support networks by affecting local structures, as these local structures may also influent the development of peer support networks. For example, van Rijsewijk et al. (2020) explored help networks and found that reciprocity can affect the formation of helping ties, showing that adolescents are likely to obtain help from each other. Some researchers studied other networks and found other important local structures in the development of networks, such as “transitive reciprocated triplets” for friendship networks (Rambaran et al., 2019). Additionally, the development of peer support networks is beneficial for the health of network members. People in higher-density peer networks are more likely to be healthier (Almquist, 2011; Babarro et al., 2016), and high activity can reduce the risk of mental illness and improve healthy behaviors and academic achievement (Loprinzi and Joyner, 2016; Wright et al., 2017; van Rijsewijk et al., 2018). Here, high activity means that a person has many relationships in a group (Lusher and Robins, 2013). In summary, researchers can gain insights into peer support relationships and networks by exploring the developmental process of peer support networks. Researchers can then seek ways to promote such networks and improve the development of their members.

## Role of Friendships in the Development of Peer Support Networks

Exploring the factors that impact peer support networks can help researchers understand the influence of factors and seek methods to improve the development of networks. According to sensitive interactions systems theory (Barbee and Cunningham, 1995), the characteristics of support seekers and providers, such as personality and social skills, and the type of relationship between them will affect support relationships between them. Previous studies explored the roles of some kinds of relationships. For example, organizational psychologists found that trust relationships mediate collaborations and social support relationships in a co-working network (Bianchi et al., 2018). Developmental psychologists explored the process of social support in networks of college students and found that accessibility between students plays an important role in dyadic support relationships (Small and Sukhu, 2016). Previous studies showed that friendships can improve peer support in various ways (Barbee and Cunningham, 1995), underscoring the importance of exploring the role of friendships in the development of peer support networks.

Friendships play an important role in the development of adolescents and provide support in the interpersonal process. Adolescents derive a sense of personal connections from the process of sharing emotions and behaviors with friends. Friendships then enhance adolescents’ sense of well-being and protect them from problems within the peer group (Bukowski et al., 2009). Therefore, friendships can provide support to youths, and youths can receive comfort from friendships (Bukowski et al., 2009). Friendships can also help adolescents receive support in another way. Adolescents seek and receive support from friends when they are in trouble (D’Avanzo et al., 2012; Heerde and Hemphill, 2018; Schacter and Margolin, 2019; van den Toren et al., 2020), such as academic and emotional problems (Tishby et al., 2001; Amemiya and Wang, 2017; Zander et al., 2019). This tendency can be explained in several ways. First, people may feel embarrassment or fear of rejection when they seek support from others (Kuhlman et al., 2019; Pabian, 2019). Friendships can mitigate these negative feelings because friends are usually trustworthy and care about each other’s well-being (Hartup, 1996; Berndt and McCandless, 2009). Therefore adolescents are less embarrassed to seek support from friends. Second, adolescents are familiar with their friends (Bukowski et al., 2009), so they know well which friends are capable of providing support to solve problems. This allows adolescents to choose proper supporters among their friends. Third, support providers have to devote time and resources when they support others, but the providers may perceive fewer costs when they help a friend (McGuire, 2003). Therefore, dyadic friendships promote peer support relationships between two adolescents.

Adolescents usually study and play in peer groups, and networks reflect all relationships among members in groups. Researchers could investigate the role of friendships in a group by exploring the influence of friendship networks on peer support networks. Researchers could use multiplex network methods to explore this cross-network issue. Previous studies of other networks have shown ways to analyze relationships between two networks. For example, Rambaran et al. (2015) found that the formation and maintenance of antipathic ties are affected by multi-person friendships in school groups by exploring relationships between friendship networks and antipathy networks. van Rijsewijk et al. (2020) recently investigated the interplay between adolescents’ friendships and the exchange of help and identified the contribution of dyadic friendships to the formation of help relationships. Unknown, however, are the effects of multi-person friendships on the development of peer support networks.

In addition, the present study explored peer support networks in classrooms from a support-seeking perspective for several reasons. First, most Chinese junior high school students are organized in the same classrooms and spend much time in classrooms, so classmates become an important source of peer support. Thus, it is necessary to explore peer support networks in classrooms. Second, students often face academic and daily problems in classrooms, and they usually seek support from peers to solve problems (e.g., Zander et al., 2019). Third, peer support that is sought from others is more likely to occur according to the support seeker’s needs (Yan, 2018), which has practical significance.

## Research Questions of the Present Study

### Role of Reciprocity and Transitivity in Peer Support Networks

Previous studies showed that reciprocity and transitivity are important features of the dynamic process of social support (Isherwood et al., 2016; van Rijsewijk et al., 2020). Reciprocity means that two members of a social network both receive support from each other and provide support to each other (Jung, 1990; Isherwood et al., 2016). This could be explained by social resource theory and equity theory. Social resource theory suggests that people obtain social resources that they need by exchanging resources with other members in a network, such as money, information, and support (Törnblom and Kazemi, 2012). Equity theory posits that negative feelings arise when the benefits one receives do not match the contributions one makes (Pritchard, 1969). Individuals are likely to feel that they are treated unfairly when they give more than they receive, and they might feel ashamed when they receive more than they give. To avoid such feelings, people try to achieve a balance between contributions and benefits (Bowling et al., 2005). Researchers have found reciprocity in networks of received peer support (van Rijsewijk et al., 2020). We further assume that if a student seeks and receives support from a classmate, then the classmate is expected to seek support from the student in the future (see Hypothesis 1 in Table 1).

**TABLE 1:** Hypotheses of the present study.

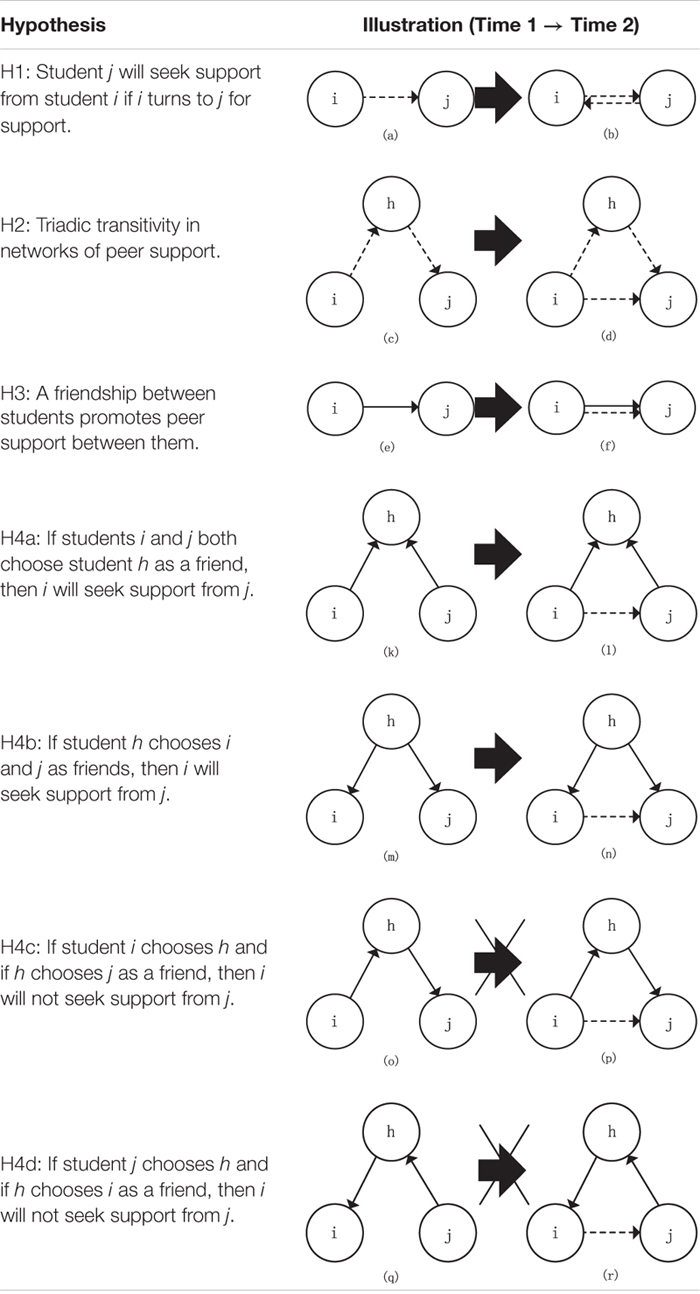

*Friendships are represented by solid lines, and peer support relationships are represented by dashed lines.*

Transitivity in a network represents a tendency for hierarchical path closure (Lusher and Robins, 2013). One common form of transitivity in a social support network is triadic transitivity, which means that a peer support tie is likely to form if there is an indirect tie between them. Researchers have found triadic transitivity in networks of received peer support (van Rijsewijk et al., 2020). In the present study, triadic transitivity was also assumed to be an important configuration in the peer support network (see Hypothesis 2 in Table 1). In other words, if student *i* seeks support from *h* and if *h* seeks support from *j*, then *i* is more likely to seek support from *j*. This may be because *h* knows the needs of *i* and the ability of *j* and then introduces *i* to seek support from *j*.

### Influence of Friendships on Peer Support Relationships

In the present study, relationships between friendship networks and peer support networks were first analyzed using Moran’s I autocorrelation and Jaccard index. Afterward, the roles of dyadic and triadic friendships in the development of peer support networks were explored by longitudinal multiplex social network analysis. A recent study carefully analyzed the contribution of friendships to the formation and maintenance of peer support ties from the perspective of received support (van Rijsewijk et al., 2020). The present study evaluated the role of friendships from the perspective of support-seeking, with the assumption that friendships may still play a positive role in the formation of support ties (see Hypothesis 3 in Table 1).

The role of triadic friendships was analyzed in detail by investigating the role of a mutual friend. A mutual friend is very common in triadic friendships, in which one individual is a friend of the other two individuals at the same time (Rambaran et al., 2019; Ripley et al., 2020). For example, student *h* is a mutual friend of classmates *i* and *j* if *h* is a friend of both *i* and *j*. A mutual friend may play a special role in the process of peer support between two adolescents. People always turn for support to someone who is both accessible and capable of providing support (van der Rijt et al., 2013; Cavallo and Hirniak, 2017; Zander et al., 2019). When two people share a mutual friend, they are more likely to meet and spend time together with this friend (Block, 2015). This process may promote accessibility and familiarity between two people and in turn contribute to the formation of peer support. Therefore, we hypothesized that a student would affect the formation of peer support ties between his/her two friends in a classroom (see Hypothesis 4 in Table 1).

Several studies of friendship networks have found that a mutual friend plays various roles in the formation of a new friendship between his/her two friends when the directions of friendships are considered (Franken et al., 2017; Simone et al., 2018; Gremmen et al., 2019). More specifically, if individual *i* chooses *h* and if *h* chooses *j* as a friend, then *i* is more likely to choose *j* as a new friend but less likely vice versa. This result prompted us to include the direction of friendship in the present study. Because the direction partly implies social status and support capabilities, the directions of friendships may influence peer support relationships. First, students tend to seek support from classmates who have more support sources (Törnblom and Kazemi, 2012). Considering the findings that the recipient of one friendship usually has a high social status, which usually means more social resources (Hallinan, 1978; Ball and Newman, 2013; Dijkstra et al., 2013; Yu and Xie, 2017), the recipient is more likely to have adequate supportive resources to be a supporter (Song et al., 2011). Meanwhile, people like to choose others who have some attractive features as friends, such as athletic talent, good social skills, and outstanding academic performance (Kubitschek and Hallinan, 1998; Taylor et al., 2006). Some of these features are also important considerations when students choose classmates from whom they seek support (Zander et al., 2019; van den Toren et al., 2020). As a result, students are more likely to seek support from recipients of friendships. In the present study, this means that support ties are likely to form when two students are chosen as supporters by the same classmate because the two students are likely both capable of providing support. Second, a large status gap may be detrimental to the formation of support ties. A student is likely to be reluctant to seek support from classmates who have much higher social status than himself/herself because of embarrassment and fear of rejection (Kuhlman et al., 2019; Pabian, 2019). Students who have a much higher status are also less likely to seek support from classmates who have a much lower status because these classmates tend to have fewer resources to help others. Therefore, a large status gap may hinder the formation of peer support ties. And peer support ties between *i* and *j* are less likely to form if *i* chooses *h* and if *h* chooses *j* as friends.

As discussed above, it is meaningful to distinguish initiators and recipients of friendships when we explore the development process of peer support. Thus, Hypothesis 4 can be divided into four parts (Table 1, H4a–H4d):

H4a: If students *i* and *j* both choose student *h* as a mutual friend, then *i* will seek support from *j*.
H4b: If student *h* chooses *i* and *j* as friends, then *i* will seek support from *j*.
H4c: If student *i* chooses *h* and if *h* chooses *j* as a friend, then *i* will not seek support from *j.*
H4d: If student *j* chooses *h* and if *h* chooses *i* as a friend, then *i* will not seek support from *j*.

### Role of Sex in Peer Support Networks and Friendship Networks

Considering the important role of sex in the development of peer relationships, we explored the role of sex in peer support networks and friendship networks. Studies of adolescents have explored the sex differences of adolescents in peer relationships. Some studies have found that boys have more friends than girls (Gest et al., 2007), while other studies have shown the opposite results (Rose and Rudolph, 2006; Zheng, 2010). With regard to social support, girls appear to receive, provide and seek more social support (Turner and Marino, 1994; Levpušček, 2006), as they have higher levels of self-disclosure (Bauminger et al., 2008). Despite this, few studies have explored the sex differences of peer support or friendship relationships among adolescents in classrooms. The present study adds to the extant literature by exploring the numbers of relationships of students in peer support networks and friendship networks, as well as examining the sex differences of these relationships.

Sex Segregation is common in peer groups among adolescents. Previous studies have shown that children and adolescents prefer to associate with the same sex (Shrum et al., 1988; Kriesi and Imdorf, 2019), implying adolescents are more likely to seek support from the same sex. However, few researchers have explored the influence of same-sex or different-sex dyads on the development of peer support networks or friendship networks. Furthermore, considering triadic relationships are important configurations in these networks, the effects of triads of the same sex and mixed ones should be explored. Therefore, there is a need to explore the sex effects of dyads and triads in peer support networks and friendship networks.

In summary, the present study should explore the role of sex in the development of peer support networks and friendship networks. In furthering this research agenda, two research questions were proposed:

Q1: Are there sex differences in the number of ties and sex segregation in peer support networks and friendship networks?
Q2: How do the effects of dyads and triads of the same-sex and mixed-sex influence the development of peer support networks?

## Materials and Methods

To prove the hypothesis proposed and examine the research questions of this study, two waves of supporter nomination and friend nomination were used to establish the networks. The network characteristics between two school years and the dynamics of peer support networks were examined to identify the developmental features of peer support networks. We also explored the role of sex effects in peer support networks and friendship networks. At last, we explored the role of friendships in the development of peer support networks by analyzing the influence of dyadic and triadic friendships on peer support ties.

### Participants

In the present study, classrooms were drawn from two waves of the Longitudinal Research on Chinese Children’s Social Development program that were collected in April 2016 and April 2017. This program aims to explore the social development of children and adolescents in China. A total of 39 classrooms from six junior high schools were selected. All of the classrooms participated in the two waves (grade 8 at wave 1 and grade 9 at wave 2). None of the classrooms were divided or combined into new classrooms. Therefore, the stability of the classrooms ensured that most of the students did not have to transfer to other classrooms during the two school years. This is a prerequisite for longitudinal social network analysis to examine the influence of friendship networks on peer support networks (Ripley et al., 2020).

An additional set of selection criteria were applied to the 39 classrooms (Block, 2015). First, the number of students in a classroom had to be ≥ 15; otherwise, the size of a network would be too small to obtain good parameter estimates (Ripley et al., 2020). Second, each network should contain some stable ties over two waves for parameter estimation (Ripley et al., 2020). Third, missing values in each network had to be sufficiently low because the stochastic actor-oriented model cannot accommodate too much missing data (Huisman and Steglich, 2008; Ripley et al., 2020). According to these criteria, 11 classrooms were dropped (three classrooms did not have enough stable ties [Jaccard index < 0.1 over two waves], and eight classrooms’ missingness was > 25%). The final sample consisted of 28 classrooms with 913 students (50.49% boys). The average classroom size was 32.61 students. All the participants were in 8th grade (mean age: 14.13 years) at the first wave and 9th grade at the second wave. A total of 2.52% of the students did not finish the questionnaire at the first wave, and 12.81% did not finish the questionnaire at the second wave. To include more classrooms in this study, we used networks in which the missing values were less than 25% (28 classrooms). Supplementary Table S3 shows the results that only included 23 classrooms (750 students) in which missing values were less than 20%. These tables show that the results of most effects under the two criteria of missing values were similar and stable.

### Procedure

Every assessment occurred in the classrooms during regular school hours. Research assistants first gave a brief introduction of the assessment to the students. The students then completed the questionnaire in paper form, including self-reports and peer nominations. During the survey, research assistants were present to answer questions when necessary. All of the assistants were psychology or education students who had received training in applying the surveys.

At the beginning of the questionnaire, the students read the informed consent after they received a full explanation of the study and its procedures. They then signed the consent form if they would like to participate in the study. Students were assured of the confidentiality of their responses, and they could quit the survey at any time. Because all of the students were minors, we obtained consent from their parents before the survey. All of the surveys and procedures complied with the ethical principles of the American Psychological Association.

### Measures

#### Peer Support Networks

Peer support networks were measured with peer nominations for peer support. In both waves, the students were asked, “Do you ask your classmates for help when you have worries or problems? Who do you ask for help?” The students were presented with a classroom list with the names and IDs of their classmates, and they responded by entering an unlimited number of classmate IDs. The nomination item was revised from previous studies that investigated social support networks (Frost et al., 2016; Lee et al., 2016; Wright et al., 2017). These nominations were translated into matrices, in which 1 indicated supporter nomination and 0 indicated non-nomination between students. We could then generate peer support networks that consisted of students for each classroom.

#### Friendship Networks

Friendship networks were measured with friend nominations (Geven et al., 2017; Kisfalusi et al., 2019; Rambaran et al., 2019). They were asked to indicate their friends by answering the question, “Who are your friends in your classroom?” This question was asked in each wave (Yuan, 2013). The students responded with the classmate IDs of their friends, and they could choose as many classmates as they wanted. Afterward, friend nominations were translated into matrices, in which 1 indicated friend nomination and 0 indicated non-nomination between two classmates, resulting in directed friendship networks that consisted of all students for each classroom.

## Analytic Strategy

### Descriptive Analyses

To describe the characteristics of peer support networks, several network indices were computed by R package “sna” (Butts, 2019). These indices include density, at least one out-tie, at least one in-tie, average degree, SD outdegree, SD indegree, asymmetric ties, mutual ties, triadic ties, and the triadic transitivity index. In support networks, density shows the overall supportive connection in a classroom. At least one out-tie means the proportion of students who choose at least one classmate to seek support, and at least one in-tie means the proportion of students who provide support to at least one classmate. These two indices could be used as isolated indices, as a student who has no tie is the isolated point in a network. Average degree shows the average number of support ties that per student has in a classroom. SD outdegree and SD indegree are the standard deviations of outdegree and indegree, respectively. A high SD outdegree (SD indegree) means the vast inequality of supportive out-ties (in-ties) among students in a classroom. Triadic ties show the number of triads that are connected. The triadic transitivity index reflects the proportion of triads in which a student chooses a supporter’s supporter. Change indicators and the Jaccard index between two waves were then obtained by the “Rsiena” package (see the overview of Rsiena below; Ripley et al., 2020). The change indicators reflect the change in the number of ties in a network, including creating ties, dissolving ties, and stable ties. The Jaccard index shows the stability of a network.

To describe the relationships between friendship networks and peer support networks, the Jaccard index was used to examine the overlap between them. Moran’s I autocorrelation was calculated by the “ape” package to explore whether friends have a similar number of out-ties or in-ties in peer support networks (Paradis et al., 2020).

To describe the role of sex in the number of ties and percentages of dyadic ties, the R package “sna” (Butts, 2019) and an R program edited by the authors were used to calculate the number of ties of boys/girls and the number of dyadic ties according to their sexes. Then the sex-differences of the numbers of out-ties and in-ties were calculated by *t*-test in SPSS 20.0.

### Longitudinal Multiplex Social Network Analysis

A longitudinal multiplex social network analysis was used to analyze the developmental process of networks, which is a kind of Simulation Investigation for Empirical Network Analysis (SIENA; Ripley et al., 2020). This method is implemented according to the stochastic actor-oriented model, allowing researchers to examine the development of friendships and peer support simultaneously and the co-evolution between the two networks (Snijders et al., 2007). Several studies have used this method to explore relationships between two different types of networks, such as disliking and status (Pál et al., 2016), bullying and defending (Huitsing et al., 2014), and friendship and bullying (Rambaran et al., 2019).

The longitudinal multiplex social network analysis in the present study consisted of two steps. In the first step, network effects were analyzed for each classroom separately, and the model specification for each classroom was the same. In the second step, the results of all classrooms were combined by employing meta-analysis in R software (Snijders and Baerveldt, 2003; Ripley et al., 2020).

To choose the proper sex effects for the final model, model 0 was used in exploratory research to find out the role of sex in the networks (to answer Q2). And then, the proper sex effects were included in model 1 (the final model) to test H1 to H4. In model 1, some classrooms were not initially convergent; thus, we fixed some effects (see details in Supplementary Table S1), and then all of the models showed good convergence (see details in Supplementary Table S2; Ripley et al., 2020). The majority of the models fit well (see details in Supplementary Table S2). All 28 classrooms were used in the final meta-analysis model to ensure representativeness. Supplementary Table S4 shows the results without classrooms that had poor Goodness of Fit, indicating that most results of the effects were the same and stable. Detailed information about the Goodness of Fit indices is presented in Supplementary Table S2.

#### Model Specification (Model 0): The Sex Effects

We first explored the role of sex in peer support networks and friendship networks. The results could also help to determine which sex effects should be included in model 1. The computation of models for each classroom was performed using RSiena 1.2-24 (Ripley et al., 2020). In the current models, the rate effects and structural effects were controlled [see details in *Model specification (model 1): the development of peer support networks*] to obtain unbiased estimates (Snijders et al., 2013). And then we test the sex effect. The “Same X” is a dyadic covariate effect that reflects the tendency that people choose members with the same covariate (X) value. The “different X” is a dyadic covariate effect that reflects the tendency that people choose members with a different covariate (X) value. The “transitive triplets same X” is a triadic covariate effect that reflects the number of transitive triplets *i*→*h*→*j*←*i* when student *i* and *j* have the same covariate (X) value. The “transitive triplets different X” is a triadic covariate effect that reflects the number of transitive triplets *i*→*h*→*j*←*i* when student *i* and *j* have a different covariate (X) value. The “transitive triplets homogeneous X” is a triadic covariate effect that reflects the number of transitive triplets *i*→*h*→*j*←*i* when *i*, *j* and *h* have the same covariate (X) value. The “transitive triplets jumping to different X” is a triadic covariate effect that reflects the number of transitive triplets *i*→*h*→*j*←*i* when *i* and *h* have the same covariate (X) value, but *j* has a different covariate (X) value. These effects were tested in model 0 (as shown in Table 3), and the significant effects would be included in model 1. Because the covariance matric was not positive definite when all the triadic effects were included in one model, we estimated the four triadic effects in four models, respectively (see details of the four models, i.e., model 0A to 0D, in Supplementary Material). And the results showed that only the Same X effect was significant in peer support networks.

#### Model Specification (Model 1): The Development of Peer Support Networks

The computation of models for each classroom was performed using R package for SIENA (RSiena 1.2-24; Ripley et al., 2020), which yields three types of effects: rate effects, structural effects, and cross-network effects. Rate effects reflect the rate of change between waves for each network (Ripley et al., 2020). Structural effects reflect structural changes in a network. We used these effects to explore the developmental process of peer support networks. Meanwhile, to obtain unbiased estimates of the effects of friendship on peer support relationships, some effects of friendship networks were also controlled (Snijders et al., 2013). The structural effects that were included in the network model for each classroom were “outdegree,” “reciprocity,” “indegree popularity,” “outdegree activity,” “number of distances two,” and some triadic effects and quaternary effects. “Outdegree” reflects the number of nominations in a network. “Reciprocity” reflects the tendency of two people to nominate each other. “Indegree popularity” reflects the tendency of students with high nominations to attract extra nominations. “Outdegree activity” means that students who nominate more classmates will nominate more in the future. “Number of distances two” means the number of classmates to whom a student is indirectly tied. Transitive triplets means that the existence of ties *i*→*h* and *h*→*j* contribute to the formation of tie *i*→*j*. “Transitive reciprocity triplets” implies that the transitive triplet improves the formation of tie *i*↔*j* (Ripley et al., 2020). Sex segregation is a powerful phenomenon during adolescence. Therefore, the Same Sex effect was included in friendship and peer support networks.

#### Model Specification (Model 1): The Role of Friendship

Cross-network effects were used to explore the role of friendship in the development of peer support networks. For these effects, the network in the role of the dependent variable is denoted by X, and the network in the role of the independent variable is denoted by W (Ripley et al., 2020). We tested the influence of the friendship network (network W) on the peer support network (network X). The “Effect of W on X” indicates that the existence of a tie from student *i* to student j in network W (friendship network) promotes the creation or maintenance of a tie from *i* to *j* in network X (peer support network; see Table 1, [e]→[f]), and this effect would test Hypothesis 3. The “agreement along W leading to X effect” indicates that when two students (*i* and *j*) nominate the same student, *h*, in network W (as a friend), *i* is more likely to nominate j in network X as a supporter (see Table 1, [k]→[l]). The “closure of shared incoming WW ≥ X effect” indicates that the shared incoming ties in network W (friendship: *h*→*i* and *h*→*j*) contribute to tie *i*→*j* in network X (peer support network; see Table 1, [m]→[n]). The “mixed WW ≥ X closure effect” indicates that students are likely to have ties in network X (supporting ties: *i*→*j*) with those whom they have a transitive tie in network W (friendship: *i*→*h*→*j*; see Table 1, [o]→[p]). The “mixed cyclic WW ≥ X closure effect” indicates that students are likely to create a tie in network X (*i*→*j* in peer support network) with those from whom they receive an indirect *tie* in network W (*j*→*h*→*i* in friendship network; see Table 1, [q]→[r]). These four effects would test Hypothesis 4.

Some researchers may argue that the influence of a mutual friend on peer support is based on the formation of a new friendship. In other words, two students who share a mutual friend will become friends first, and then the friendship would improve peer support between them. For example, Block (2015) suggested that if two people have a mutual friend, then they are more likely to meet and spend time together and thus become friends. In fact, the influence of a new friendship on peer support relationships is controlled when the effect of a dyadic friendship on peer support is included in the model. Thus, we could explore the influence of a mutual friend on peer support directly, regardless of the influence of a mutual friend on the formation of a new friendship.

Finally, because some students were not at school in one wave, missingness was treated as noninformative data in the estimation process (Huisman and Steglich, 2008).

## Results

### Descriptive Results

#### Characteristics of Peer Support Networks

Table 2 shows the summarized descriptive results for the 28 classrooms. Part 1 of Table 2 presents structural indices for the two kinds of networks in each wave. The descriptive statistics of peer support networks are shown in the fourth and fifth columns. The density of the networks shows that the networks were very sparse in both waves (0.07 and 0.06, respectively). More than 70% of the students had chosen at least one supporter (0.74 and 0.73 in the two waves, respectively), and more than 75% of the students had been chosen as supporters by their classmates (0.85 and 0.77, respectively). On average, a student had about two supporters from whom they could seek support (2.23 and 1.92). The out-ties were more uneven than the in-ties, demonstrated by the SD degree. The dyadic indicators showed many more asymmetric ties (41.54 and 38.36) than mutual ties (15.82 and 12.32). The triadic indicators also showed that triadic relationships were common in peer support networks.

**TABLE 2:** Descriptive statistics of friendship networks and peer support networks.

Part 1: Per time points	Friendship network		Peer support network	
	Wave 1	Wave 2	Wave 1	Wave 2
**Density indicators**				
Density^a^	0.20 (0.15, 0.33)	0.16 (0.08, 0.30)	0.07 (0.04, 0.10)	0.06 (0.03, 0.11)
**Number of ties**				
At least one out-tie^b^	0.95 (0.85, 1.00)	0.90 (0.48, 1.00)	0.74 (0.61, 0.91)	0.73 (0.52, 0.91)
At least one in-tie^c^	0.99 (0.91, 1.00)	0.95 (0.83, 1.00)	0.85 (0.70, 1.00)	0.77 (0.59, 0.94)
Average degree^d^	6.24 (4.69, 8.35)	5.08 (2.33, 8.34)	2.23 (1.38, 3.09)	1.92 (0.97, 3.69)
SD outdegree	4.51 (2.83, 7.38)	4.37 (2.56, 7.94)	2.05 (1.26, 3.26)	2.01 (1.32, 3.74)
SD indegree	2.67 (1.54, 3.66)	2.53 (1.42, 3.50)	1.64 (0.99, 2.19)	1.60 (1.01, 2.59)
**Dyadic indicators**				
Asymmetric ties	94.39 (57, 158)	81.54 (41, 141)	41.54 (25, 60)	38.36 (18, 63)
Mutual ties	54.61 (30, 87)	42.46 (4, 71)	15.82 (6, 29)	12.32 (2, 33)
Reciprocity^e^	0.54 (0.42, 0.66)	0.50 (0.11, 0.71)	0.42 (0.29, 0.61)	0.38 (0.11, 0.52)
**Triadic indicators**				
Triadic ties^f^	932.68 (351, 2101)	654.79 (240, 1173)	159.61 (53, 321)	132.79 (39, 429)
Transitivity index^g^	0.51 (0.37, 0.65)	0.47 (0.30, 0.60)	0.36 (0.21, 0.60)	0.33 (0.17, 0.56)
**The role of sex^h^**				
Number of out-ties of boy	7.15*** (0, 34)	6.01*** (0, 39)	2.04** (0, 17)	1.65*** (0, 16)
Number of out-ties of girl	5.74 (0, 37)	4.59 (0, 39)	2.45 (0, 13)	2.22 (0, 13)
Number of in-ties of boy	6.67* (0, 16)	5.39 (0, 15)	1.86*** (0, 8)	1.48*** (0, 7)
Number of in-ties of girl	6.24 (0, 16)	5.19 (0, 17)	2.64 (0, 10)	2.39 (0, 10)
Boy → boy	0.45 (0.11, 0.72)	0.43 (0.11, 0.61)	0.40 (0.06, 0.61)	0.35 (0.09, 0.61)
Boy → girl	0.10 (0.02, 0.20)	0.12 (0.01, 0.26)	0.07 (0.00, 0.24)	0.09 (0.00, 0.18)
Girl → boy	0.07 (0.01, 0.16)	0.08 (0.01, 0.29)	0.03 (0.00, 0.13)	0.05 (0.00, 0.16)
Girl → girl	0.38 (0.16, 0.81)	0.36 (0.13, 0.85)	0.50 (0.27, 0.92)	0.51 (0.33, 0.88)

**Part 2: Between time points**	**Friendship Network Wave 1→Wave 2**		**Peer Support Network Wave 1→Wave 2**	

**Change indicators**				
Creating ties (0→1)	64.75 (28, 111)		32.54 (13, 84)	
Dissolving ties (1→0)	81.50 (48, 137)		34.89 (13, 55)	
Stable ties (1→1)	96.25 (41, 138)		28.79 (13, 45)	
Jaccard index^i^	0.39 (0.20, 0.54)		0.30 (0.20, 0.42)	

**Part 3: Between networks**	**Friendship × Support seeking**		**Friendship × Support providing**	
**Per time point**	**Wave 1**	**Wave 2**	**Wave 1**	**Wave 2**

**Dependence indicators**				
Moran’s I autocorrelation^j^	0.06 (−0.20, 0.27)	0.13 (−0.10, 0.63)	0.28 (−0.16, 0.80)	0.30 (−0.18, 0.78)
Jaccard index^k^	0.31 (0.15, 0.50)	0.33 (0.19, 0.57)	—	—

*The table shows the average. The range of indices is presented in parentheses.*

*^*a*^Density is the number of observed ties divided by the total number of possible ties.*

*^*b*^At least one out-tie is calculated as the number of students who had at least one out-tie divided by the total number of students in a classroom.*

*^*c*^At least one in-tie was calculated as the number of students who had at least one in-tie divided by the total number of students in a classroom.*

*^*d*^Average degree means the average number of indegrees in a friendship (or peer support) network.*

*^*e*^Reciprocity is the proportion of mutual connections. In this study, it was calculated as 2M / (2M + A), where M is the number of mutual ties, and A is the number of asymmetric ties in a network.*

*^*f*^ Triadic ties were the number of triads in which one was connected with the other.*

*^*g*^Transitivity index means the probability that the adjacent students of a student were connected.*

^*h*^The number of ties means the average number of out/in ties of boys or girls, and * means the significance of differences between boys and girls where *p ≤ 0.05, **p ≤ 0.01, ***p ≤ 0.001 (two-tailed test); boy/girl → boy/girl means the percentage of ties that boys/girls chosen boys/girls.

*^*i*^The Jaccard index is the proportion of stable ties to create ties and dissolve ties.*

*^*j*^Moran’s I autocorrelation measures the association between friendship and support seeking (outdegree) or support providing (indegree).*

*^*k*^The Jaccard index means the proportion of overlap between friendship networks and peer support networks.*

Part 2 of Table 2 evaluated changes in networks between the two waves. Many ties were created, dissolved, and maintained in peer support networks. The Jaccard index (0.30) also showed that peer support networks had certain stability and changeability.

#### Relationship Between Friendship Networks and Peer Support Networks

Part 1 of Table 2 shows all of the indices of friendship networks were larger than peer support networks, implying that peer support networks were sparser than friendship networks. Part 2 of Table 2 shows that more ties were created, dissolved, and maintained in friendship networks than in peer support networks, and friendship networks were more stable than peer support networks (Jaccard indices = 0.39 vs. 0.30).

Part 3 of Table 2 shows a close relationship between the two networks. Moran’s I autocorrelation calculates the relationship between friendship and individual support conditions (Moran, 1950; Steglich et al., 2010; Butts, 2019). The range of values of Moran’s I is [−1, 1]. Values that are close to 0 imply that friends are unrelated to support (referring to random pairing). Values that are close to 1 imply that friends are similar in support seeking or providing. Values that are close to −1 imply that friends are different in support seeking or providing. Ordinarily, values up to 0.2 show clear behavioral similarity (Steglich et al., 2010; Rambaran et al., 2019). In the present study, moderate values of Moran’s I between friendship and support providing (0.28 and 0.32) indicated modest similarity in support providing for friends in each wave, and the similarity between friendship and support seeking was smaller. Values of the Jaccard index between friendship networks and peer support networks showed moderate overlap (0.31 and 0.33), indicating that students often sought support from some of their friends.

#### The Role of Sex

Part 1 of Table 2 also shows the role of sex in peer support networks and friendship networks. Boys had fewer out-ties (2.04^∗∗^) and in-ties (1.86^∗∗∗^) than girls (2.45 and 2.64) in wave 1, and girls still had more out-ties (2.22) and in-ties (2.39) than boys (1.65^∗∗∗^ and 1.48^∗∗∗^) in wave 2. These results implied girls chose more supporters in classrooms, and girls were chosen as supporters by more classmates. In friendship networks, however, boys chose more friends in both waves (7.15^∗∗∗^ and 6.01^∗∗∗^) than girls (5.74 and 4.59), and boys were chosen by more classmates as friends in wave 1 (6.67^∗^ vs. 6.24). Sex segregation is obvious in both networks in each wave. In peer support networks, more than 85% (90% and 86%) of students sought support from the same sex in each wave. In friendship networks, about 80% (83% and 79%) of students chose same-sex classmates as friends. Therefore, Q1 was answered.

### Rsiena Estimates of Dynamic Networks

#### Analysis of the Sex Effects

Table 3 presents the Rsiena results of the sex effects. In peer support networks, the positive same-sex effect (1.02) indicated that students are more likely to seek support from same-sex classmates. The different-sex effect was not significant, implying that the difference of sex had no significant effect on the dyadic support ties. None of the four sex effects of triads (transitive triplets same/different/homogeneous/jumping sex) was significant, implying that the same sex or mixed sex of the triads did not affect support ties. Therefore, the Q2 was answered.

**TABLE 3:** Results from Rsiena analysis that test the role of sex.

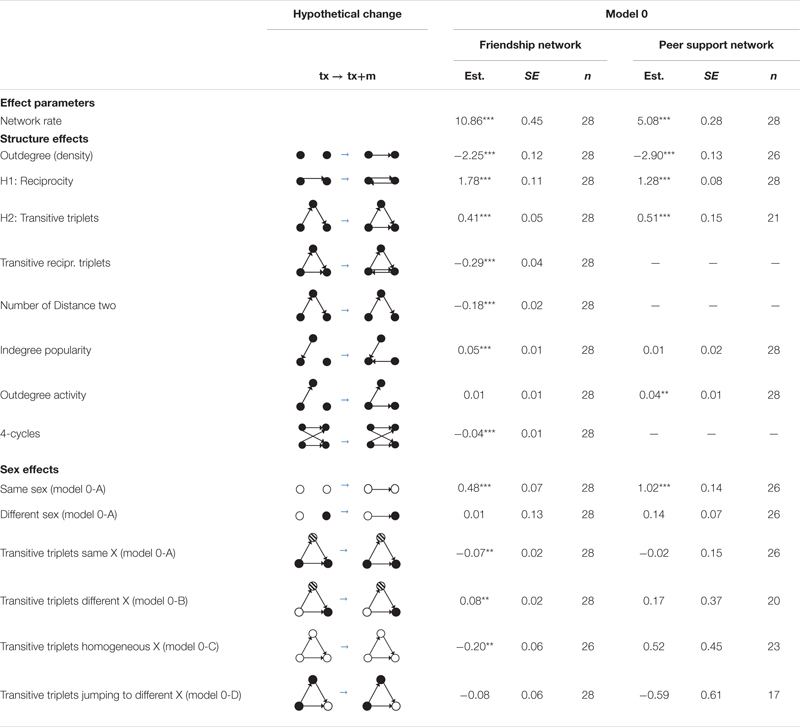

Significance was tested by dividing the estimates with the standard error, resulting in *t*-values that were approximately normally distributed (Ripley et al., 2020). Convergence statistics: *t*-ratio all < 0.10, and overall maximum convergence ratio < 0.25 (see details in Supplementary Tables S0A2, S0B2, S0C2, S0D2 for Model 0). *p ≤ 0.05, **p ≤ 0.01, ***p ≤ 0.001 (two-tailed test).

Additionally, in friendship networks, the positive same-sex effect (0.48) indicated that students were more likely to choose same-sex classmates as friends. The negative estimates of the transitive triplets same X effect (Est. = −0.07, *p* < 0.01) and transitive triplets homogeneous X effect (Est. = −0.20, *p* < 0.01) showed that these two kinds of triadic relationships were less likely formed in friendship networks. The positive estimate of the transitive triplets different X effect (Est. = 0.08, *p* < 0.01) showed that student *i* was likely to choose an opposite-sex classmate *j* as a friend when *i* chose *h* and if *h* chose *j* as a friend.

#### Dyadic Change in Peer Support Networks

Table 4 presents the Rsiena results. The positive network rate (4.09) indicated that peer support networks changed significantly over time. The positive reciprocity effect (1.07) and transitive triplets effect (0.30) in peer support networks verified hypotheses 1 and 2. A student would seek support from a classmate if this classmate sought support from the student earlier. A student was also likely to seek support from his/her supporter’s supporter. The indegree popularity effect was not significant, implying that a student would not attract more support seekers if this student had several support seekers earlier. The positive outdegree activity effect indicates that a student would seek support from more classmates if this student sought support earlier, but the value of the effect was very small (0.08). Additionally, peer support ties were more likely formed between the same sex, indicated by the same-sex effect (0.39).

**TABLE 4:** Results from RSiena analysis that predicted the influence of friendships on peer support.

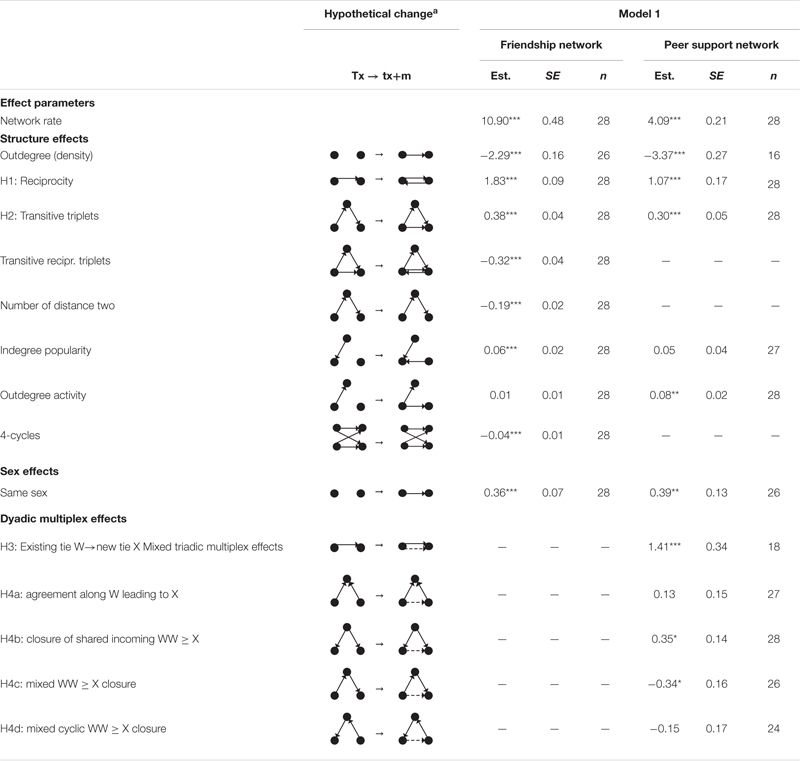

Significance was tested by dividing the estimates with the standard error, resulting in *t*-values that were approximately normally distributed (Ripley et al., 2020). Convergence statistics: *t*-ratio all < 0.10, and overall maximum convergence ratio < 0.25 (see details in Supplementary Table S2 for Model 1). ^*a*^Friendships are represented by solid lines, and peer support relationships are represented by dashed lines when there two kinds of lines in the diagrams. *p ≤ 0.05, **p ≤ 0.01, ***p ≤ 0.001 (two-tailed test).

#### Influence of Friendship on Peer Support

Peer support from friends was tested as a dyadic multiplex effect. Table 4 shows that the mean estimate of the Effect of W on X was positive and significant (Est. = 1.41, *p* < 0.001), implying that the initiator of a friendship tended to seek support from the recipient. This was consistent with the results of the influence of friendships on received peer support (van Rijsewijk et al., 2020).

The influence of a mutual friend on peer support was tested as four mixed triadic multiplex effects (see Table 1, H4a–d). The hypotheses were partially supported. A peer support tie between two students was likely to form if they were chosen as friends by the same classmate (see Model 1 in Table 4, closure of shared incoming effect, Est. = 0.35, *p* < 0.05), but when they chose the same person as a friend, it seemed the mutual friend had no significant effect on the peer support tie (agreement along W leading to X effect, Est. = 0.13, *p* > 0.05). The negative estimate of the mixed WW ≥ X closure effect (Est. = −0.34, *p* < 0.05) showed that student *i* was unlikely to seek support from classmate *j* if *i* chose *h* and if *h* chose *j* as a friend. However, the estimate of the mixed cyclic WW ≥ X closure effect was nonsignificant (Est. = −0.15, *p* > 0.05). In summary, only hypotheses 4b and 4c were proven.

## Discussion

The dynamic development of peer support networks was researched in a sample of adolescents in 28 middle school classrooms. We assumed that peer support networks develop during adolescence, and friendship plays multiple roles in the development of peer support networks. These hypotheses were partially proven by the longitudinal multiplex social network analysis, but unexpected results were also found.

### Sparsity, Hierarchy, and Sex Segregation of Peer Support Networks

Peer support networks are very sparse networks, in which each student only chose an average of about two classmates. This could be explained by sensitive interactions systems theory (Barbee and Cunningham, 1995). When students have problems, support seekers must consider several factors before they seek support from supporters, such as the characteristics of the seeker and supporter and the relationship between them. Therefore, choosing appropriate supporters might not be easy. Because of embarrassment or fear of rejection, people usually choose familiar and trustworthy people from whom they can seek support (Bianchi et al., 2018; Kuhlman et al., 2019; Pabian, 2019). Supporters should also be capable of solving support seekers’ problems (Törnblom and Kazemi, 2012). As a result, very few people meet these requirements, thus explaining sparse peer support networks. Previous studies showed that the number of supporters is important for mental health (Wright et al., 2017; Santini et al., 2019), so the sparsity of peer support networks is unfavorable to students.

Peer support networks are hierarchical networks, in which some students have more support ties while others have much fewer ties. We found that the SD of outdegrees was roughly equal to the average degree, implying an unbalanced distribution of support-seeking ties. This is consistent with previous studies and theories of relationships among peers within peer groups. Although relationships among peers are considered to be equal relationships relative to hierarchical relationships between adolescents and parents (Bukowski et al., 2009), peers are not always equal within peer groups (Hawley, 1999). This could be explained by theories of social dominance. Adolescents can gain social status by acts of leadership, cooperation, and aggression within groups (Hawley, 1999). Therefore, students with higher social status are usually more skillful in seeking and providing support, whereas others with lower social status may be reluctant to seek support (Barbee and Cunningham, 1995). Consistent with this, 27% of the students did not seek support from classmates, and more than 15% of the students were not supporters from whom others sought support. This suggests that educators should facilitate the ability of students to seek support and lower the number of isolated adolescents in peer support networks. This would be beneficial for students’ development.

Peer support networks are largely segregated by sex. This is consistent with previous studies that showed that children and adolescents prefer to associate with the same sex (Shrum et al., 1988; Kriesi and Imdorf, 2019). Students are more likely to seek support from familiar people, and they naturally ask for support from same-sex classmates. This also implies that many students tend to not seek support from half of their classmates, which appears to not be beneficial for the development of peer support networks. The results also indicate that girls have more support ties in peer support networks, and this is consistent with prior studies (Turner and Marino, 1994; Levpušček, 2006). But we found boys have more friendship ties than girls, and this result verifies the point of view that boys’ friendship networks are larger than girls’ (Gest et al., 2007). Additionally, some same-sex and mixed-sex effects were significant in friendship networks. The “transitive triplets same X” effect was negative. This may be because we have included the dyadic same-sex effect in the model, and then the dyadic same-sex effect influenced the “transitive triplets same X” effect. We have tried to remove the same-sex effect and found the “transitive triplets same X” effect became positive. The “transitive triplets different X” effect was positive, implying *i* was more likely to choose *j* as a friend if *i* chose *h* and *h* chose *j* as a friend. The two effects show that *i* is more likely to choose *j* as a friend if they have a mutual friend *h*, regardless of the sex of *i* and *j.* But “transitive triplets homogeneous X” effect was negative, showing there were fewer triads of the same sex compared to the random numbers of the triads in networks. This is consistent with the fact that not all students are sex-segregated in friendship networks.

### Development of Peer Support Networks

In the present study, both reciprocity and transitivity effects improved the formation of peer support ties. Reciprocity is common in the process of peer support. For example, an earlier study identified a reciprocity effect in support networks from a received support perspective (van Rijsewijk et al., 2020). The present study further showed that reciprocity is important for the formation of support ties from a support-seeking perspective. This could be explained by social contagion theory and social resource theory. Social contagion theory suggests that group members influence peers’ mental health and behaviors (Friedman, 2004; Eisenberg et al., 2013). According to this theory, students’ support-seeking behaviors are influenced by classmates’ support-seeking behaviors, and therefore, students are more likely to seek support if others sought support from them earlier. Social resource theory also suggests that people would exchange support resources in their social environment (Törnblom and Kazemi, 2012). Additionally, the present results indicated that support-seeking ties from seekers were also beneficial for support providers. Earlier findings showed that peer support not only is beneficial for support seekers but also allows supporters to enhance their own self-esteem and improve healthy function (Pierce et al., 1996). The present study found that providing support improved providers’ support seeking, in which providers were likely to seek support from their own support seekers. In summary, the reciprocity effect showed that support-seeking was beneficial for both seekers and providers.

The significance of transitivity proves that the support tie between two students is affected by their classmates, and one may seek support from the supporter’s supporter. This can be explained by resource theory (Törnblom and Kazemi, 2012). Student *h* usually seeks support from classmate *j* who has resources to supply help. If another classmate, *i*, seeks support from *h* but *h* is incapable of helping *i*, then *h* would introduce *j* who has support resources to help *i*. Additionally, the indegree popularity effect is not significant, and the effect size of outdegree activity is very small, showing that the two effects are unimportant for the development of peer support networks. In summary, reciprocity and transitivity effects could improve the formation of peer support ties, thereby improving the development of peer support networks.

### Diversity of Friendship Roles

The present study partially confirmed the assumption that friendships improve the formation of peer support ties. The result that dyadic friendship relationships positively affected the formation of dyadic support relationships is consistent with previous theories of the relationship between friendship and support seeking (Barbee and Cunningham, 1995; van den Toren et al., 2020). Furthermore, a kind of triadic friendship improved peer support relationships. This result could be attributable to the peer support process and social resource views (Song et al., 2011). When two students were chosen as friends by the same classmate, they were both recipients in their friendships (H4b) and likely had high status in the group (Hallinan, 1978). This implies that they may be both attractive in the classroom because of good academic performance, social skills, emotional abilities, or other characteristics. Therefore, they are more likely capable of supporting classmates who are in trouble. Because they share a mutual friend, they have more opportunities to spend time together and get acquainted with each other (Block, 2015). Therefore, the capability of offering support and familiarity would improve the formation of peer support ties.

Under some conditions, however, friendship may hinder the formation of peer support ties. When student *i* chooses *h* and *h* chooses *j* as a friend, there may be a gap between students *i* and *j*. In this triadic friendship, student *j* who has a higher status is likely to be more attractive and capable of helping others, whereas student *i* who has a lower status is likely to be less attractive and capable. The results of H4c in Table 4 show that although student *j* is likely to have supportive resources to help others, student *i* still does not tend to seek support from student *j*. This could be interpreted by the gap of social status between them. The gap of social status leads to student *i* being reluctant to seek support from *j* because of fear of rejection or awkwardness (Kim and Yon, 2019; Pabian, 2019). This suggests that although a mutual friend could improve familiarity between two classmates, the social status gap could hinder the formation of peer support ties.

Friendships sometimes have no significant influence on support ties. When two students choose the same classmate as a friend, they are both initiators of their friendship (H4a) and likely have lower status in the group (Hallinan, 1978). This suggests that they are likely incapable of providing support when classmates are in trouble. As a result, even if they share a mutual friend who helps them become familiar, they do not seek support from the other side more. Similarly, the mutual friend does not encourage student *j* to seek support from student *i* when *i* chooses *h* and *h* chooses *j* as friends (as shown in H4d in Table 4). This is probably because student *i* who lies at the end of the friendship is not sufficiently capable of supporting the others, and then student *j* does not need to seek support from *i*.

Overall, although friendships play an important role in the development of peer support networks, other factors likely affect the role of friendships. Under the influences of these factors, friendships could play different roles in the formation of support ties.

### Possible Role of Culture

Previous studies explored the interplay between received support relationships and friendship in Dutch adolescents. Some of the results from this Western study are different from the present study. First, we found that the average number of supporters for a student in our study (2.23 and 1.92, respectively) was smaller than in the peer support networks in Dutch adolescents (>2.45 in all waves; van Rijsewijk et al., 2020), although the average size of the networks in our study was larger (32.33 vs. 23.24). Earlier studies showed that people from collectivistic countries are less likely to turn to others for support because they are taught not to disturb others for personal reasons (Taylor et al., 2004; Kim et al., 2006). This may explain the fewer supporters per student in peer support networks among Chinese students. Second, the average effect size (size = 1.11, odds equal to *e*^1.11^ = 3.03, Table 4) of reciprocity in networks in the present study was much larger than the effect size (size = 0.30, odds equal to *e*^0.30^ = 1.35) in the Dutch networks (van Rijsewijk et al., 2020). This could be explained by the argument that people always consider ingroup members as much more important than outgroup members in collectivistic cultures compared with individualistic cultures, so there are more support relationships between ingroup members (Aronson et al., 2019), thus resulting in more mutual ties within a group. Therefore, students are more likely to support each other in a collectivistic culture. Another explanation is based on the traditional Chinese ethics principle of Baoen. This is one of the basic morals and behavioral patterns in traditional Chinese culture, emphasizing that people who are helped by others should be grateful for the favor and repay it to help providers when providers are in trouble (Liu, 2012). In this context, support seekers know they should help supporters in the future when they need it. Thus, providers could seek and receive support easily from former seekers, and then reciprocity is much higher in peer support networks among Chinese adolescents.

Considering that the support networks in the Dutch study consisted of receiving support ties, the differences between support seeking and support receiving may also contribute to the different results between studies. First, students sometimes receive support from classmates, although they did not seek support. Therefore, the number of ties that are received is possibly larger than the ties that are sought. Second, according to social resource theory and equity theory (Pritchard, 1969; Törnblom and Kazemi, 2012), if a student seeks support from a classmate, then the classmate is more likely to seek support reciprocally from seekers after offering support. When a classmate proactively provides support to a student, some of the voluntary support may be inappropriate (Pierce et al., 1996; Silverstein et al., 1996), and then the support receiver would not return support in this situation. Therefore, researchers should investigate seeking and receiving networks in various cultures and compare the results to confirm the role of culture in the development of peer support networks.

### Implications

The present findings have several important theoretical and practical implications for the development of adolescents’ peer support relationships and networks. First, it is important to expand the field of relationship research both ecologically and systematically. Ecological systems theory (Bronfenbrenner and Morris, 2007) emphasizes the role of the environment in the development of individuals. Group socialization theory (Harris, 1995) suggests that peer groups contribute to the development of personality and behaviors. The present study found that both individual characteristics and dyadic relationships were influenced by environmental factors. Thus, environmental factors further affect the development of networks. These results illustrate the necessity of expanding ecological systems theory and group socialization theory to research on the development of interpersonal relationships and investigate interactions among individual characteristics, relationships between them, and features of the environment.

Second, teachers could improve peer support networks by reducing the number of isolated students and sex segregation within networks. As shown in the present study, more than 25% of the students did not seek support from their classmates. Teachers could analyze the reasons with these students, such as a fear of rejection (Kim and Yon, 2019; Pabian, 2019) or being unaware of their problems (Barbee and Cunningham, 1995), and encourage these students to seek support from classmates. Additionally, students usually seek support from students of the same sex, implying that they do not seek support from about half of their classmates. To reduce the influence of sex segregation on peer support, teachers could improve familiarity and interpersonal relationships between boys and girls (Mehta and Strough, 2009) by organizing class activities in various forms.

Third, students should be aware that they have to do something (such as working hard) to attract friends and make themselves capable of supporting others. They would then be more likely to have more opportunities and confidence to seek and receive support from others (Barbee and Cunningham, 1995). Dyadic friendships are beneficial for the development of support ties. If students could improve their attractiveness to make more friends, then they would be more likely to have supporters. The complex roles of mutual friends imply that some factors that are associated with friendships play active roles in the development of peer support ties. Therefore, parents could help their children improve some types of abilities, such as emotional skills, motor skills, and leadership (Törnblom and Kazemi, 2012). Such abilities and social status could then help them lower their fear of rejection when they seek peer support (Kim and Yon, 2019; Pabian, 2019).

### Limitations and Directions for Future Research

Some limitations of the present study should be considered. First, based on earlier studies of social support that people seek from others (Frost et al., 2016; Lee et al., 2016; Wright et al., 2017), the item that we used in the questionnaire in this study meant that a student receives support from classmates when they seek support from them. This is reasonable because students in the 8th and 9th grades are familiar with their classmates, and they are more likely to seek support from classmates who are capable of providing and willing to provide support. Some researchers are still concerned that people may not receive support through support seeking and thus added the phrase “get support” in the item (Melkman and Benbenishty, 2018). The addition of this phrase may be a more rigorous way to explore support networks. Future research should compare networks that are measured by the two statements to further explore whether they should be distinguished.

Second, by incorporating the direction of peer support and friendship in classroom networks, we could test more intricate hypotheses about the dynamic influence of friendship on peer support. However, we do not exactly know the role of individual characteristics in these processes. Researchers have found that specific characteristics of support seekers and providers influence the process of peer support (Barbee and Cunningham, 1995; Dujardin et al., 2016; Cavallo and Hirniak, 2017; Pabian, 2019). For example, although we found that students did not tend to seek support from classmates when they chose the same friends, the peer support tie may form if we include their support resources into the model. More detailed information about support seekers and providers (e.g., academic achievement and emotional ability) may allow a better understanding of the developmental process of peer support networks.

Third, we found that the dynamic characteristics of peer support networks and various roles of friendship influenced the development of peer support networks in Chinese culture. Based on these results, researchers could further explore the characteristics of peer support networks and the ways in which friendship influences the development of these networks in other cultures. By comparing the results in various cultures, researchers could further systematically analyze the ways in which friendship and culture influence the development of peer support networks.

## Conclusion

In summary, the present study explored the developmental process of peer support networks among adolescents in China. Researchers could investigate multi-person relationships and overall characteristics of peer support relationships in groups according to peer support networks. We found a sparsity, hierarchy, and sex segregation of peer support networks and identified dyadic and triadic features of support relationships. We also found sex differences both in the participation of peer support networks and friendship networks. The effects of reciprocity and transitivity played important roles in the development of peer support networks. A mutual friend affected peer support ties between the other two students in various ways, based on the direction of friendships. These findings illustrate the developmental process of peer support networks and the role of friendship in this process, further demonstrating the importance of studying peer support networks and considering the complex role of friends in the developmental process of support networks.

## Data Availability Statement

The raw data supporting the conclusions of this article will be made available by the authors, without undue reservation.

## Ethics Statement

The studies involving human participants were reviewed and approved by the Ethics Review Committee of Beijing Normal University. Written informed consent to participate in this study was provided by the participants’ legal guardian/next of kin.

## Author Contributions

LW designed and executed the study, analyzed the data, and wrote the manuscript. LL collaborated in designing the research. ZL collaborated in manuscript revisions. KY assisted with designing the research and manuscript revisions. JJ assisted with designing the research. YB provided overall guidance. All authors contributed to the article and approved the submitted version.

## Conflict of Interest

The authors declare that the research was conducted in the absence of any commercial or financial relationships that could be construed as a potential conflict of interest.
