# Supplementary material for: The Developmental Process of Peer Support Networks: The Role of Friendship
Source: Front Psychol. 2021 Jan 28;12:615148. doi: 10.3389/fpsyg.2021.615148 (PMC7875894; doi:10.3389/fpsyg.2021.615148)
Supplement: Supplementary file 1 [file Table_1.DOCX]

Supplementary Materials：

**Table S1:** Effects included in all SIENA multivariate network models summarized in Table 3

**Table S2:** Goodness of Fit statistics for SIENA multivariate network models summarized in Table 3

**Table S3** Results from RSiena analysis that predicted the influence of friendships on peer support (23 classrooms (20% missing value))

**Table S4** Results from RSiena analysis that predicted the influence of friendships on peer support (17 classrooms, without classrooms having poor GOF)

*Because the covariance matric of the model was not positive definite when all the triadic effects were included in one model, we estimated the four triadic effects in four models respectively (see details of the four models in Table S0A3, Table S0B3, Table S0C3, and Table S0D3 respectively). Some classrooms were not initially convergent; thus, we fixed some effects (see details in Table S0A1 to S0D1), and then all of the models showed good convergence (see details in Table S0A2 to S0D2; Ripley et al., 2020). The majority of the models fit well (see details in Table S0A2 to S0D2). Table S0A5 to S0D5 shows the results without classrooms that had poor Goodness of Fit, indicating that most results of the effects were the same and stable. Detailed information about the Goodness of Fit indices is presented in Table S0A2 to S0D2.*

**Table S0A1:** Effects included in all SIENA multivariate network models 0-A summarized in Table 3

**Table S0A2:** Goodness of Fit statistics for SIENA multivariate network models 0-A summarized in Table 3

**Table S0A3:** Results from RSiena analysis that test the role of sex 0-A (28 classrooms)

**Table S0A4:** Results from RSiena analysis that test the role of sex 0-A (23 classrooms (20% missing value))

**Table S0A5:** Results from RSiena analysis that test the role of sex 0-D (17 classrooms, without classrooms having poor GOF)

**Table S0B1:** Effects included in all SIENA multivariate network models 0-B summarized in Table 3

**Table S0B2:** Goodness of Fit statistics for SIENA multivariate network models 0-B summarized in Table 3

**Table S0B3:** Results from RSiena analysis that test the role of sex 0-B (28 classrooms)

**Table S0B4:** Results from RSiena analysis that test the role of sex 0-B (23 classrooms (20% missing value))

**Table S0B5:** Results from RSiena analysis that test the role of sex 0-D (16 classrooms, without classrooms having poor GOF)

**Table S0C1:** Effects included in all SIENA multivariate network models 0-C summarized in Table 3

**Table S0C2:** Goodness of Fit statistics for SIENA multivariate network models 0-C summarized in Table 3

**Table S0C3:** Results from RSiena analysis that test the role of sex 0-C (26 classrooms)

**Table S0C4:** Results from RSiena analysis that test the role of sex 0-C (21 classrooms (20% missing value))

**Table S0C5:** Results from RSiena analysis that test the role of sex 0-C (16 classrooms, without classrooms having poor GOF)

**Table S0D1:** Effects included in all SIENA multivariate network models 0-D summarized in Table 3

**Table S0D2:** Goodness of Fit statistics for SIENA multivariate network models 0-D summarized in Table 3

**Table S0D3:** Results from RSiena analysis that test the role of sex 0-D (28 classrooms)

**Table S0D4:** Results from RSiena analysis that test the role of sex 0-D (23 classrooms (20% missing value))

**Table S0D5:** Results from RSiena analysis that test the role of sex 0-D (17 classrooms, without classrooms having poor GOF)

**Table S1** Effects included in all SIENA multivariate network models summarized in Table 3

| **Friendship Networks** | 1 | 2 | 3 | 4 | 5 | 6 | 7 | 8 | 9 | 10 | 11 | 12 | 13 | 14 | 15 | 16 | 17 | 18 | 19 | 20 | 21 | 22 | 23 | 24 | 25 | 26 | 27 | 28 |
| --- | --- | --- | --- | --- | --- | --- | --- | --- | --- | --- | --- | --- | --- | --- | --- | --- | --- | --- | --- | --- | --- | --- | --- | --- | --- | --- | --- | --- |
| *Rate Effects* |  |  |  |  |  |  |  |  |  |  |  |  |  |  |  |  |  |  |  |  |  |  |  |  |  |  |  |  |
| Network rate t1-t2 |  |  |  |  |  |  |  |  |  |  |  |  |  |  |  |  |  |  |  |  |  |  |  |  |  |  |  |  |
| *Structure Effects* |  |  |  |  |  |  |  |  |  |  |  |  |  |  |  |  |  |  |  |  |  |  |  |  |  |  |  |  |
| Outdegree (density) |  |  |  |  |  |  |  |  |  |  |  |  |  |  |  |  |  |  |  |  |  |  |  |  |  |  |  |  |
| Reciprocity |  |  |  |  |  |  |  |  |  |  |  |  |  |  |  |  |  |  |  |  |  |  |  |  |  |  |  |  |
| Transitive triplets |  |  |  |  |  |  |  |  |  |  |  |  |  |  |  |  |  |  |  |  |  |  |  |  |  |  |  |  |
| Transitive reciprocated triplets |  |  |  |  |  |  |  |  |  |  |  |  |  |  |  |  |  |  |  |  |  |  |  |  |  |  |  |  |
| Number of distance 2 |  |  |  |  |  |  |  |  |  |  |  |  |  |  |  |  |  |  |  |  |  |  |  |  |  |  |  |  |
| Indegree popularity |  |  |  |  |  |  |  |  |  |  |  |  |  |  |  |  |  |  |  |  |  |  |  |  |  |  |  |  |
| Outdegree activity |  |  |  |  |  |  |  |  |  |  |  |  |  |  |  |  |  |  |  |  |  |  |  |  |  |  |  |  |
| Four-cycles |  |  |  |  |  |  |  |  |  |  |  |  |  |  |  |  |  |  |  |  |  |  |  |  |  |  |  |  |
| *Sex Effects* |  |  |  |  |  |  |  |  |  |  |  |  |  |  |  |  |  |  |  |  |  |  |  |  |  |  |  |  |
| Same-sex |  |  |  |  |  |  |  |  |  |  |  |  |  |  |  |  |  |  |  |  |  |  |  |  |  |  |  |  |
| **Support Networks** | 1 | 2 | 3 | 4 | 5 | 6 | 7 | 8 | 9 | 10 | 11 | 12 | 13 | 14 | 15 | 16 | 17 | 18 | 19 | 20 | 21 | 22 | 23 | 24 | 25 | 26 | 27 | 28 |
| *Rate Effects* |  |  |  |  |  |  |  |  |  |  |  |  |  |  |  |  |  |  |  |  |  |  |  |  |  |  |  |  |
| Network rate t1-t2 |  |  |  |  |  |  |  |  |  |  |  |  |  |  |  |  |  |  |  |  |  |  |  |  |  |  |  |  |
| *Structure Effects* |  |  |  |  |  |  |  |  |  |  |  |  |  |  |  |  |  |  |  |  |  |  |  |  |  |  |  |  |
| Outdegree (density) |  |  |  |  |  |  |  |  |  |  |  |  |  |  |  |  |  |  |  |  |  |  |  |  |  |  |  |  |
| Reciprocity |  |  |  |  |  |  |  |  |  |  |  |  |  |  |  |  |  |  |  |  |  |  |  |  |  |  |  |  |
| Transitive triplets |  |  |  |  |  |  |  |  |  |  |  |  |  |  |  |  |  |  |  |  |  |  |  |  |  |  |  |  |
| Indegree popularity |  |  |  |  |  |  |  |  |  |  |  |  |  |  |  |  |  |  |  |  |  |  |  |  |  |  |  |  |
| Outdegree activity |  |  |  |  |  |  |  |  |  |  |  |  |  |  |  |  |  |  |  |  |  |  |  |  |  |  |  |  |
| *Sex Effects* |  |  |  |  |  |  |  |  |  |  |  |  |  |  |  |  |  |  |  |  |  |  |  |  |  |  |  |  |
| Same-sex |  |  |  |  |  |  |  |  |  |  |  |  |  |  |  |  |  |  |  |  |  |  |  |  |  |  |  |  |
| *Dyadic Multiplex Effects* |  |  |  |  |  |  |  |  |  |  |  |  |  |  |  |  |  |  |  |  |  |  |  |  |  |  |  |  |
| Existing tie W → new tie X |  |  |  |  |  |  |  |  |  |  |  |  |  |  |  |  |  |  |  |  |  |  |  |  |  |  |  |  |
| *Mixed Triadic Multiplex Effects* |  |  |  |  |  |  |  |  |  |  |  |  |  |  |  |  |  |  |  |  |  |  |  |  |  |  |  |  |
| From tie W → agreement tie X |  |  |  |  |  |  |  |  |  |  |  |  |  |  |  |  |  |  |  |  |  |  |  |  |  |  |  |  |
| Tie W → closure agreement tie X |  |  |  |  |  |  |  |  |  |  |  |  |  |  |  |  |  |  |  |  |  |  |  |  |  |  |  |  |
| Tie W → cyclic closure tie X |  |  |  |  |  |  |  |  |  |  |  |  |  |  |  |  |  |  |  |  |  |  |  |  |  |  |  |  |
| Shared tie W → incoming tie X |  |  |  |  |  |  |  |  |  |  |  |  |  |  |  |  |  |  |  |  |  |  |  |  |  |  |  |  |

*All models were analyzed using 10,000 iterations for better convergence and reliability of the parameter estimates and standard errors. In some classrooms, the estimates and standard errors of some effects were very high, and the t-ratios for convergence are very large. As a possible solution, some effects were fixed at a non-zero value (based on score-type tests) by trial and error to get better convergence (Ripley et al., 2020). Fixed parameters are indicated with black cells. Gray cells indicate effects estimated freely.*

| **Table S2** Goodness of Fit statistics for SIENA multivariate network models summarized in Table 3 | | | | | | | | | |
| --- | --- | --- | --- | --- | --- | --- | --- | --- | --- |
|  | | Friendship Networks | | | | Peer Support network | | | |
| Classroom | Overall convergence | Outdegree distribution | Indegree distribution | Geodesic distance | Triad census | Outdegree distribution | Indegree distribution | Geodesic distance | Triad census |
| 1 | 0.19 | 0.294 | 0.254 | 0.216 | 0.971 | 0.773 | 0.702 | 0.378 | 0.514 |
| 2 | 0.16 | 0.098 | 0.218 | 0.158 | 0.001 | 0.330 | 0.400 | 0.371 | 0.286 |
| 3 | 0.17 | 0.478 | 0.759 | 0.425 | 0.898 | 0.230 | 0.830 | 0.026 | 0.338 |
| 4 | 0.14 | 0.946 | 0.967 | 0.718 | 0.999 | 0.797 | 0.186 | 0.825 | 0.444 |
| 5 | 0.13 | 0.069 | 0.271 | 0.830 | 0.809 | 0.350 | 0.579 | 0.135 | 0.056 |
| 6 | 0.20 | 0.143 | 0.371 | 0.530 | 0.131 | 0.837 | 0.653 | 0.997 | 0.610 |
| 7 | 0.19 | 0.102 | 0.990 | 0.959 | 0.265 | 0.848 | 0.832 | 0.818 | 0.008 |
| 8 | 0.15 | 0.182 | 0.290 | 0.992 | 0.262 | 0.376 | 0.441 | 0.659 | 0.935 |
| 9 | 0.16 | 0.005 | 0.873 | 0.232 | 0.001 | 0.517 | 0.730 | 0.358 | 0.509 |
| 10 | 0.17 | 0.199 | 0.450 | 0.172 | 0.153 | 0.450 | 0.994 | 0.542 | 0.999 |
| 11 | 0.13 | 0.422 | 0.410 | 0.796 | 0.473 | 0.079 | 0.512 | 0.207 | 0.558 |
| 12 | 0.16 | 0.139 | 0.581 | 0.089 | 0.809 | 0.038 | 0.152 | 0.538 | 0.178 |
| 13 | 0.16 | 0.001 | 0.219 | 0.956 | 0.677 | 0.150 | 0.975 | 0.248 | 0.499 |
| 14 | 0.13 | 0.410 | 0.658 | 0.608 | 1.000 | 0.946 | 0.741 | 0.469 | 0.906 |
| 15 | 0.16 | 0.099 | 0.667 | 0.829 | 0.397 | 0.084 | 0.237 | 0.739 | 0.393 |
| 16 | 0.13 | 0.246 | 0.680 | 0.588 | 0.310 | 0.466 | 0.758 | 0.558 | 0.508 |
| 17 | 0.17 | 0.250 | 0.544 | 0.863 | 0.956 | 0.528 | 0.908 | 0.990 | 0.411 |
| 18 | 0.16 | 0.005 | 0.495 | 0.970 | 0.000 | 0.063 | 0.996 | 0.891 | 0.010 |
| 19 | 0.13 | 0.074 | 0.916 | 0.594 | 0.443 | 0.345 | 0.728 | 0.889 | 0.846 |
| 20 | 0.17 | 0.871 | 0.031 | 0.096 | 0.288 | 0.000 | 0.619 | 0.924 | 0.775 |
| 21 | 0.14 | 0.983 | 0.577 | 0.344 | 0.927 | 0.756 | 0.188 | 0.528 | 0.472 |
| 22 | 0.13 | 0.895 | 0.953 | 0.254 | 0.007 | 0.912 | 0.087 | 0.139 | 0.306 |
| 23 | 0.12 | 0.819 | 0.369 | 0.667 | 0.943 | 0.165 | 0.694 | 0.391 | 0.538 |
| 24 | 0.14 | 0.744 | 0.907 | 0.569 | 0.64 | 0.346 | 0.797 | 0.706 | 0.248 |
| 25 | 0.16 | 0.106 | 0.142 | 0.244 | 0.227 | 0.060 | 0.056 | 0.941 | 0.102 |
| 26 | 0.14 | 0.509 | 0.672 | 0.029 | 0.000 | 0.914 | 0.155 | 0.358 | 0.098 |
| 27 | 0.17 | 0.37 | 0.038 | 0.060 | 0.468 | 0.466 | 0.849 | 0.979 | 0.049 |
| 28 | 0.14 | 0.085 | 0.727 | 0.489 | 0.517 | 0.109 | 0.663 | 0.891 | 0.965 |
| *Goodness of fit (GoF) was assessed with four computed auxiliary network statistics: out-degree distribution, in-degree distribution, geodesic distance, and triad census. Poor GOF (<0.05) are indicated with grey cells.* | | | | | | | | | |

**Table S3** Results from RSiena analysis that predicted the influence of friendships on peer support (23 classrooms: 20% missing value)

|  | Hypothetical change | | |  | Model1 | | | | | | |
| --- | --- | --- | --- | --- | --- | --- | --- | --- | --- | --- | --- |
|  |  |  |  |  | Friendship network | | |  | Peer Support network | | |
|  | tx | → | tx+m |  | Est. | SE | n |  | Est. | SE | n |
| Effect parameters |  |  |  |  |  |  |  |  |  |  |  |
| Network rate |  |  |  |  | 10.95^***^ | 0.52 | 23 |  | 4.02^***^ | 0.23 | 23 |
| Structure effects |  |  |  |  |  |  |  |  |  |  |  |
| Outdegree (density) |  | **→** |  |  | -2.27^***^ | 0.18 | 21 |  | -3.25^***^ | 0.28 | 14 |
| Reciprocity |  | **→** |  |  | 1.84^***^ | 0.10 | 23 |  | 1.09^***^ | 0.20 | 23 |
| Transitive triplets |  | **→** |  |  | 0.38^***^ | 0.05 | 23 |  | 0.33^***^ | 0.06 | 23 |
| Transitive recipr. triplets |  | **→** |  |  | -0.31^***^ | 0.04 | 23 |  | —— | —— | —— |
| Number of Distance two |  | **→** |  |  | -0.20^***^ | 0.02 | 23 |  | —— | —— | —— |
| Indegree popularity |  | **→** |  |  | 0.05^*^ | 0.02 | 23 |  | 0.00 | 0.03 | 22 |
| Outdegree activity |  | **→** |  |  | 0.00 | 0.01 | 23 |  | 0.06^*^ | 0.03 | 22 |
| 4-cycles |  | **→** |  |  | -0.04^***^ | 0.01 | 23 |  | —— | —— | —— |
| Sex effects |  |  |  |  |  |  |  |  |  |  |  |
| Same-sex |  | **→** |  |  | 0.38^***^ | 0.07 | 23 |  | 0.39^*^ | 0.15 | 21 |
| Dyadic multiplex effects |  |  |  |  |  |  |  |  |  |  |  |
| H1:Existing tie W → new tie X |  | **→** |  |  | —— | —— | —— |  | 1.29^**^ | 0.36 | 16 |
| Mixed triadic multiplex effects |  |  |  |  |  |  |  |  |  |  |  |
| H2a: agreement along W  leading to X |  | **→** |  |  | —— | —— | —— |  | 0.09 | 0.18 | 22 |
| H2b: closure of shared  incoming WW=>X |  | **→** |  |  | —— | —— | —— |  | 0.36^*^ | 0.16 | 23 |
| H2c: mixed WW=>X closure |  | **→** |  |  | —— | —— | —— |  | -0.34^+^ | 0.19 | 21 |
| H2d: mixed cyclic WW=>X  closure |  | **→** |  |  | —— | —— | —— |  | -0.10 | 0.19 | 19 |

*Significance was tested by dividing the estimates with the standard error, resulting in t values that were approximately normally distributed (Ripley et al., 2020). Convergence statistics: t-ratio all < 0.10, and overall maximum convergence ratio < 0.25. ^a^Friendships are represented by solid lines, and peer support relationships are represented by dashed lines. ^+^p ≤ 0.10, *p ≤ 0.05, **p ≤ 0.01, ***p ≤ 0.001 (two-tailed test).*

**Table S4** Results from RSiena analysis that predicted the influence of friendships on peer support (17 classrooms without classrooms having poor GOF)

|  | Hypothetical change | | |  | Model1 | | | | | | |
| --- | --- | --- | --- | --- | --- | --- | --- | --- | --- | --- | --- |
|  |  |  |  |  | Friendship network | | |  | Peer Support network | | |
|  | tx | → | tx+m |  | Est. | SE | n |  | Est. | SE | n |
| Effect parameters |  |  |  |  |  |  |  |  |  |  |  |
| Network rate |  |  |  |  | 10.36^***^ | 0.56 | 17 |  | 4.11^***^ | 0.26 | 17 |
| Structure effects |  |  |  |  |  |  |  |  |  |  |  |
| Outdegree (density) |  | **→** |  |  | -2.17^***^ | 0.16 | 16 |  | -3.72^***^ | 0.35 | 10 |
| Reciprocity |  | **→** |  |  | 1.86^***^ | 0.13 | 17 |  | 1.00^***^ | 0.22 | 17 |
| Transitive triplets |  | **→** |  |  | 0.40^***^ | 0.04 | 17 |  | 0.28^**^ | 0.08 | 17 |
| Transitive recipr. triplets |  | **→** |  |  | -0.38^***^ | 0.05 | 17 |  | —— | —— | —— |
| Number of Distance two |  | **→** | **** |  | -0.19^***^ | 0.03 | 17 |  | —— | —— | —— |
| Indegree popularity |  | **→** |  |  | 0.06^**^ | 0.02 | 17 |  | 0.11^*^ | 0.04 | 16 |
| Outdegree activity |  | **→** |  |  | 0.01 | 0.01 | 17 |  | 0.10^**^ | 0.03 | 16 |
| 4-cycles |  | **→** |  |  | -0.04^***^ | 0.01 | 17 |  | —— | —— | —— |
| Sex effects |  |  |  |  |  |  |  |  |  |  |  |
| Same-sex |  | **→** |  |  | 0.32^**^ | 0.08 | 17 |  | 0.46^*^ | 0.17 | 16 |
| Dyadic multiplex effects |  |  |  |  |  |  |  |  |  |  |  |
| H1:Existing tie W → new tie X |  | **→** |  |  | —— | —— | —— |  | 1.12^*^ | 0.46 | 10 |
| Mixed triadic multiplex effects |  |  |  |  |  |  |  |  |  |  |  |
| H2a: agreement along W  leading to X |  | **→** |  |  | —— | —— | —— |  | 0.13 | 0.18 | 16 |
| H2b: closure of shared  incoming WW=>X |  | **→** |  |  | —— | —— | —— |  | 0.35^*^ | 0.16 | 17 |
| H2c: mixed WW=>X closure |  | **→** |  |  | —— | —— | —— |  | -0.21 | 0.18 | 15 |
| H2d: mixed cyclic WW=>X  closure |  | **→** |  |  | —— | —— | —— |  | -0.19 | 0.21 | 14 |

*Significance was tested by dividing the estimates with the standard error, resulting in t values that were approximately normally distributed (Ripley et al., 2020). Convergence statistics: t-ratio all < 0.10, and overall maximum convergence ratio < 0.25. ^a^Friendships are represented by solid lines, and peer support relationships are represented by dashed lines. *p ≤ 0.05, **p ≤ 0.01, ***p ≤ 0.001 (two-tailed test).*

**Table S0A1:** Effects included in all SIENA multivariate network models 0-A summarized in Table 3

| **Friendship Networks** | 1 | 2 | 3 | 4 | 5 | 6 | 7 | 8 | 9 | 10 | 11 | 12 | 13 | 14 | 15 | 16 | 17 | 18 | 19 | 20 | 21 | 22 | 23 | 24 | 25 | 26 | 27 | 28 |
| --- | --- | --- | --- | --- | --- | --- | --- | --- | --- | --- | --- | --- | --- | --- | --- | --- | --- | --- | --- | --- | --- | --- | --- | --- | --- | --- | --- | --- |
| *Rate Effects* |  |  |  |  |  |  |  |  |  |  |  |  |  |  |  |  |  |  |  |  |  |  |  |  |  |  |  |  |
| Network rate t1-t2 |  |  |  |  |  |  |  |  |  |  |  |  |  |  |  |  |  |  |  |  |  |  |  |  |  |  |  |  |
| *Structure Effects* |  |  |  |  |  |  |  |  |  |  |  |  |  |  |  |  |  |  |  |  |  |  |  |  |  |  |  |  |
| Outdegree (density) |  |  |  |  |  |  |  |  |  |  |  |  |  |  |  |  |  |  |  |  |  |  |  |  |  |  |  |  |
| Reciprocity |  |  |  |  |  |  |  |  |  |  |  |  |  |  |  |  |  |  |  |  |  |  |  |  |  |  |  |  |
| Transitive triplets |  |  |  |  |  |  |  |  |  |  |  |  |  |  |  |  |  |  |  |  |  |  |  |  |  |  |  |  |
| Transitive reciprocated triplets |  |  |  |  |  |  |  |  |  |  |  |  |  |  |  |  |  |  |  |  |  |  |  |  |  |  |  |  |
| Number of distance 2 |  |  |  |  |  |  |  |  |  |  |  |  |  |  |  |  |  |  |  |  |  |  |  |  |  |  |  |  |
| Indegree popularity |  |  |  |  |  |  |  |  |  |  |  |  |  |  |  |  |  |  |  |  |  |  |  |  |  |  |  |  |
| Outdegree activity |  |  |  |  |  |  |  |  |  |  |  |  |  |  |  |  |  |  |  |  |  |  |  |  |  |  |  |  |
| Four-cycles |  |  |  |  |  |  |  |  |  |  |  |  |  |  |  |  |  |  |  |  |  |  |  |  |  |  |  |  |
| *Sex Effects* |  |  |  |  |  |  |  |  |  |  |  |  |  |  |  |  |  |  |  |  |  |  |  |  |  |  |  |  |
| Same-sex |  |  |  |  |  |  |  |  |  |  |  |  |  |  |  |  |  |  |  |  |  |  |  |  |  |  |  |  |
| Diff-sex |  |  |  |  |  |  |  |  |  |  |  |  |  |  |  |  |  |  |  |  |  |  |  |  |  |  |  |  |
| sameXTransTrip |  |  |  |  |  |  |  |  |  |  |  |  |  |  |  |  |  |  |  |  |  |  |  |  |  |  |  |  |
| **Support Networks** | 1 | 2 | 3 | 4 | 5 | 6 | 7 | 8 | 9 | 10 | 11 | 12 | 13 | 14 | 15 | 16 | 17 | 18 | 19 | 20 | 21 | 22 | 23 | 24 | 25 | 26 | 27 | 28 |
| *Rate Effects* |  |  |  |  |  |  |  |  |  |  |  |  |  |  |  |  |  |  |  |  |  |  |  |  |  |  |  |  |
| Network rate t1-t2 |  |  |  |  |  |  |  |  |  |  |  |  |  |  |  |  |  |  |  |  |  |  |  |  |  |  |  |  |
| *Structure Effects* |  |  |  |  |  |  |  |  |  |  |  |  |  |  |  |  |  |  |  |  |  |  |  |  |  |  |  |  |
| Outdegree (density) |  |  |  |  |  |  |  |  |  |  |  |  |  |  |  |  |  |  |  |  |  |  |  |  |  |  |  |  |
| Reciprocity |  |  |  |  |  |  |  |  |  |  |  |  |  |  |  |  |  |  |  |  |  |  |  |  |  |  |  |  |
| Transitive triplets |  |  |  |  |  |  |  |  |  |  |  |  |  |  |  |  |  |  |  |  |  |  |  |  |  |  |  |  |
| Indegree popularity |  |  |  |  |  |  |  |  |  |  |  |  |  |  |  |  |  |  |  |  |  |  |  |  |  |  |  |  |
| Outdegree activity |  |  |  |  |  |  |  |  |  |  |  |  |  |  |  |  |  |  |  |  |  |  |  |  |  |  |  |  |
| *Sex Effects* |  |  |  |  |  |  |  |  |  |  |  |  |  |  |  |  |  |  |  |  |  |  |  |  |  |  |  |  |
| Same-sex |  |  |  |  |  |  |  |  |  |  |  |  |  |  |  |  |  |  |  |  |  |  |  |  |  |  |  |  |
| Diff-sex |  |  |  |  |  |  |  |  |  |  |  |  |  |  |  |  |  |  |  |  |  |  |  |  |  |  |  |  |
| transitive triplets same X |  |  |  |  |  |  |  |  |  |  |  |  |  |  |  |  |  |  |  |  |  |  |  |  |  |  |  |  |

*All models were analyzed using 3,000 iterations for better convergence and reliability of the parameter estimates and standard errors. In some classrooms, the estimates and standard errors of some effects were very high, and the t-ratios for convergence are very large. As a possible solution, some effects were fixed at a non-zero value (based on score-type tests) by trial and error to get better convergence (Ripley et al., 2020). Fixed parameters are indicated with black cells. Gray cells indicate effects estimated freely.*

| **Table S0A2:** Goodness of Fit statistics for SIENA multivariate network models 0-A summarized in Table 3 | | | | | | | | | |
| --- | --- | --- | --- | --- | --- | --- | --- | --- | --- |
|  | | Friendship Networks | | | | Peer Support network | | | |
| Classroom | Overall convergence | Outdegree distribution | Indegree distribution | Geodesic distance | Triad census | Outdegree distribution | Indegree distribution | Geodesic distance | Triad census |
| 1 | 0.18 | 0.33 | 0.25 | 0.29 | 0.97 | 0.76 | 0.53 | 0.35 | 0.20 |
| 2 | 0.21 | 0.20 | 0.24 | 0.48 | 0.01 | 0.29 | 0.38 | 0.38 | 0.01 |
| 3 | 0.17 | 0.47 | 0.71 | 0.35 | 0.84 | 0.05 | 0.94 | 0.03 | 0.35 |
| 4 | 0.16 | 0.93 | 0.96 | 0.74 | 1 | 0.78 | 0.20 | 0.93 | 0.34 |
| 5 | 0.15 | 0.07 | 0.24 | 0.85 | 0.80 | 0.29 | 0.46 | 0.25 | 0.10 |
| 6 | 0.11 | 0.16 | 0.44 | 0.56 | 0.14 | 0.83 | 0.71 | 1.00 | 0.60 |
| 7 | 0.21 | 0.10 | 1.00 | 0.94 | 0.36 | 0.70 | 0.84 | 0.79 | 0.00 |
| 8 | 0.12 | 0.11 | 0.29 | 0.98 | 0.31 | 0.32 | 0.28 | 0.73 | 0.9 |
| 9 | 0.20 | 0.00 | 0.96 | 0.26 | 0 | 0.51 | 0.79 | 0.53 | 0.54 |
| 10 | 0.19 | 0.33 | 0.48 | 0.20 | 0.26 | 0.40 | 0.97 | 0.81 | 1.00 |
| 11 | 0.15 | 0.61 | 0.47 | 0.95 | 0.46 | 0.09 | 0.98 | 0.29 | 0.91 |
| 12 | 0.22 | 0.11 | 0.56 | 0.12 | 0.81 | 0.04 | 0.17 | 0.59 | 0.19 |
| 13 | 0.18 | 0.04 | 0.47 | 0.96 | 0.79 | 0.12 | 0.96 | 0.27 | 0.43 |
| 14 | 0.15 | 0.44 | 0.67 | 0.65 | 1 | 0.91 | 0.73 | 0.86 | 0.91 |
| 15 | 0.20 | 0.09 | 0.64 | 0.86 | 0.47 | 0.08 | 0.43 | 0.71 | 0.41 |
| 16 | 0.21 | 0.51 | 0.69 | 0.60 | 0.69 | 0.31 | 0.72 | 0.58 | 0.47 |
| 17 | 0.14 | 0.34 | 0.33 | 0.81 | 0.96 | 0.49 | 0.81 | 0.97 | 0.39 |
| 18 | 0.20 | 0.01 | 0.52 | 1 | 0 | 0.13 | 1.00 | 0.84 | 0.00 |
| 19 | 0.23 | 0.10 | 0.92 | 0.62 | 0.4 | 0.40 | 0.83 | 0.90 | 0.71 |
| 20 | 0.15 | 0.90 | 0.02 | 0.09 | 0.49 | 0 | 0.52 | 0.43 | 0.63 |
| 21 | 0.19 | 0.98 | 0.63 | 0.29 | 0.91 | 0.40 | 0.17 | 0.39 | 0.54 |
| 22 | 0.17 | 0.92 | 0.96 | 0.24 | 0.00 | 0.73 | 0.08 | 0.23 | 0.79 |
| 23 | 0.21 | 0.86 | 0.37 | 0.70 | 0.93 | 0.06 | 0.91 | 0.58 | 0.25 |
| 24 | 0.18 | 0.72 | 0.93 | 0.57 | 0.69 | 0.25 | 0.85 | 0.31 | 0.10 |
| 25 | 0.22 | 0.10 | 0.13 | 0.23 | 0.27 | 0.06 | 0.12 | 0.94 | 0.18 |
| 26 | 0.20 | 0.62 | 0.63 | 0.02 | 0 | 0.91 | 0.13 | 0.30 | 0.26 |
| 27 | 0.15 | 0.34 | 0.04 | 0.06 | 0.51 | 0.46 | 0.82 | 0.95 | 0.12 |
| 28 | 0.22 | 0.14 | 0.67 | 0.50 | 0.50 | 0.11 | 0.66 | 0.90 | 0.97 |
| *Goodness of fit (GoF) was assessed with four computed auxiliary network statistics: out-degree distribution, in-degree distribution, geodesic distance, and triad census. Poor GOF (<0.05) are indicated with grey cells.* | | | | | | | | | |

**Table S0A3:** Results from RSiena analysis that test the role of sex 0-A (28 classrooms)

|  | Hypothetical change | | |  | Model 0A | | | | | | |
| --- | --- | --- | --- | --- | --- | --- | --- | --- | --- | --- | --- |
|  |  |  |  |  | Friendship network | | |  | Peer Support network | | |
|  | tx | → | tx+m |  | Est. | SE | n |  | Est. | SE | n |
| Effect parameters |  |  |  |  |  |  |  |  |  |  |  |
| Network rate |  |  |  |  | 10.86^***^ | 0.45 | 28 |  | 5.08^***^ | 0.28 | 28 |
| Structure effects |  |  |  |  |  |  |  |  |  |  |  |
| Outdegree (density) |  | **→** |  |  | -2.25^***^ | 0.12 | 28 |  | -2.90^***^ | 0.13 | 26 |
| Reciprocity |  | **→** |  |  | 1.78^***^ | 0.11 | 28 |  | 1.28^***^ | 0.08 | 28 |
| Transitive triplets |  | **→** |  |  | 0.41^***^ | 0.05 | 28 |  | 0.51^***^ | 0.15 | 21 |
| Transitive recipr. triplets |  | **→** |  |  | -0.29^***^ | 0.04 | 28 |  | —— | —— | —— |
| Number of Distance two |  | **→** |  |  | -0.18^***^ | 0.02 | 28 |  | —— | —— | —— |
| Indegree popularity |  | **→** |  |  | 0.05^***^ | 0.01 | 28 |  | 0.01 | 0.02 | 28 |
| Outdegree activity |  | **→** |  |  | 0.01 | 0.01 | 28 |  | 0.04^**^ | 0.01 | 28 |
| 4-cycles |  | **→** |  |  | -0.04^***^ | 0.01 | 28 |  | —— | —— | —— |
| Sex effects |  |  |  |  |  |  |  |  |  |  |  |
| Same-sex |  | **→** |  |  | 0.48^***^ | 0.07 | 28 |  | 1.02^***^ | 0.14 | 26 |
| Different sex |  | **→** |  |  | 0.01 | 0.13 | 28 |  | 0.14 | 0.07 | 26 |
| transitive triplets same X |  | **→** |  |  | -0.07^**^ | 0.02 | 28 |  | -0.02 | 0.15 | 26 |

*Significance was tested by dividing the estimates with the standard error, resulting in t values that were approximately normally distributed (Ripley et al., 2020). Convergence statistics: t-ratio all < 0.10, and overall maximum convergence ratio < 0.25. *p ≤ 0.05, **p ≤ 0.01, ***p ≤ 0.001 (two-tailed test).*

**Table S0A4:** Results from RSiena analysis that test the role of sex 0-A (23 classrooms (20% missing value))

|  | Hypothetical change | | |  | Model 0A | | | | | | |
| --- | --- | --- | --- | --- | --- | --- | --- | --- | --- | --- | --- |
|  |  |  |  |  | Friendship network | | |  | Peer Support network | | |
|  | tx | → | tx+m |  | Est. | SE | n |  | Est. | SE | n |
| Effect parameters |  |  |  |  |  |  |  |  |  |  |  |
| Network rate |  |  |  |  | 10.94^***^ | 0.50 | 23 |  | 4.80^***^ | 0.24 | 23 |
| Structure effects |  |  |  |  |  |  |  |  |  |  |  |
| Outdegree (density) |  | **→** |  |  | -2.21^***^ | 0.13 | 23 |  | -2.87^***^ | 0.14 | 21 |
| Reciprocity |  | **→** |  |  | 1.79^***^ | 0.12 | 23 |  | 1.31^***^ | 0.09 | 23 |
| Transitive triplets |  | **→** |  |  | 0.40^***^ | 0.05 | 23 |  | 0.60^***^ | 0.20 | 16 |
| Transitive recipr. triplets |  | **→** |  |  | -0.29^***^ | 0.05 | 23 |  | —— | —— | —— |
| Number of Distance two |  | **→** |  |  | -0.18^***^ | 0.02 | 23 |  | —— | —— | —— |
| Indegree popularity |  | **→** |  |  | 0.04^*^ | 0.01 | 23 |  | 0.00 | 0.02 | 23 |
| Outdegree activity |  | **→** |  |  | 0.00 | 0.01 | 23 |  | 0.03 | 0.01 | 23 |
| 4-cycles |  | **→** |  |  | -0.03^***^ | 0.01 | 23 |  | —— | —— | —— |
| Sex effects |  |  |  |  |  |  |  |  |  |  |  |
| Same-sex |  | **→** |  |  | 0.47^***^ | 0.08 | 23 |  | 1.10^***^ | 0.18 | 21 |
| Different sex |  | **→** |  |  | 0.03 | 0.07 | 23 |  | 0.13 | 0.09 | 21 |
| transitive triplets same X |  | **→** |  |  | -0.06 | 0.03 | 23 |  | 0.00 | 0.19 | 21 |

*Significance was tested by dividing the estimates with the standard error, resulting in t values that were approximately normally distributed (Ripley et al., 2020). Convergence statistics: t-ratio all < 0.10, and overall maximum convergence ratio < 0.25. *p ≤ 0.05, **p ≤ 0.01, ***p ≤ 0.001 (two-tailed test).*

**Table S0A5:** Results from RSiena analysis that test the role of sex 0-A (17 classrooms, without classrooms having poor GOF)

|  | Hypothetical change | | |  | Model 0A | | | | | | |
| --- | --- | --- | --- | --- | --- | --- | --- | --- | --- | --- | --- |
|  |  |  |  |  | Friendship network | | |  | Peer Support network | | |
|  | tx | → | tx+m |  | Est. | SE | n |  | Est. | SE | n |
| Effect parameters |  |  |  |  |  |  |  |  |  |  |  |
| Network rate |  |  |  |  | 10.36^***^ | 0.53 | 17 |  | 4.95^***^ | 0.32 | 17 |
| Structure effects |  |  |  |  |  |  |  |  |  |  |  |
| Outdegree (density) |  | **→** |  |  | -2.22^***^ | 0.15 | 17 |  | -2.95^***^ | 0.17 | 16 |
| Reciprocity |  | **→** |  |  | 1.83^***^ | 0.13 | 17 |  | 1.15^***^ | 0.11 | 17 |
| Transitive triplets |  | **→** |  |  | 0.45^***^ | 0.05 | 17 |  | 0.33 | 0.17 | 14 |
| Transitive recipr. triplets |  | **→** |  |  | -0.36^***^ | 0.05 | 17 |  | —— | —— | —— |
| Number of Distance two |  | **→** |  |  | -0.18^***^ | 0.03 | 17 |  | —— | —— | —— |
| Indegree popularity |  | **→** |  |  | 0.05^*^ | 0.02 | 17 |  | 0.05^*^ | 0.02 | 17 |
| Outdegree activity |  | **→** |  |  | 0.01 | 0.01 | 17 |  | 0.06^***^ | 0.01 | 17 |
| 4-cycles |  | **→** |  |  | -0.03^**^ | 0.01 | 17 |  | —— | —— | —— |
| Sex effects |  |  |  |  |  |  |  |  |  |  |  |
| Same-sex |  | **→** |  |  | 0.51^***^ | 0.09 | 17 |  | 1.03^***^ | 0.21 | 16 |
| Different sex |  | **→** |  |  | -0.05 | 0.07 | 17 |  | 0.09 | 0.09 | 16 |
| transitive triplets same X |  | **→** |  |  | -0.08^*^ | 0.04 | 17 |  | 0.01 | 0.20 | 15 |

*Significance was tested by dividing the estimates with the standard error, resulting in t values that were approximately normally distributed (Ripley et al., 2020). Convergence statistics: t-ratio all < 0.10, and overall maximum convergence ratio < 0.25. *p ≤ 0.05, **p ≤ 0.01, ***p ≤ 0.001 (two-tailed test).*

**Table S0B1:** Effects included in all SIENA multivariate network models 0-B summarized in Table 3

| **Friendship Networks** | 1 | 2 | 3 | 4 | 5 | 6 | 7 | 8 | 9 | 10 | 11 | 12 | 13 | 14 | 15 | 16 | 17 | 18 | 19 | 20 | 21 | 22 | 23 | 24 | 25 | 26 | 27 | 28 |
| --- | --- | --- | --- | --- | --- | --- | --- | --- | --- | --- | --- | --- | --- | --- | --- | --- | --- | --- | --- | --- | --- | --- | --- | --- | --- | --- | --- | --- |
| *Rate Effects* |  |  |  |  |  |  |  |  |  |  |  |  |  |  |  |  |  |  |  |  |  |  |  |  |  |  |  |  |
| Network rate t1-t2 |  |  |  |  |  |  |  |  |  |  |  |  |  |  |  |  |  |  |  |  |  |  |  |  |  |  |  |  |
| *Structure Effects* |  |  |  |  |  |  |  |  |  |  |  |  |  |  |  |  |  |  |  |  |  |  |  |  |  |  |  |  |
| Outdegree (density) |  |  |  |  |  |  |  |  |  |  |  |  |  |  |  |  |  |  |  |  |  |  |  |  |  |  |  |  |
| Reciprocity |  |  |  |  |  |  |  |  |  |  |  |  |  |  |  |  |  |  |  |  |  |  |  |  |  |  |  |  |
| Transitive triplets |  |  |  |  |  |  |  |  |  |  |  |  |  |  |  |  |  |  |  |  |  |  |  |  |  |  |  |  |
| Transitive reciprocated triplets |  |  |  |  |  |  |  |  |  |  |  |  |  |  |  |  |  |  |  |  |  |  |  |  |  |  |  |  |
| Number of distance 2 |  |  |  |  |  |  |  |  |  |  |  |  |  |  |  |  |  |  |  |  |  |  |  |  |  |  |  |  |
| Indegree popularity |  |  |  |  |  |  |  |  |  |  |  |  |  |  |  |  |  |  |  |  |  |  |  |  |  |  |  |  |
| Outdegree activity |  |  |  |  |  |  |  |  |  |  |  |  |  |  |  |  |  |  |  |  |  |  |  |  |  |  |  |  |
| Four-cycles |  |  |  |  |  |  |  |  |  |  |  |  |  |  |  |  |  |  |  |  |  |  |  |  |  |  |  |  |
| *Sex Effects* |  |  |  |  |  |  |  |  |  |  |  |  |  |  |  |  |  |  |  |  |  |  |  |  |  |  |  |  |
| same-sex |  |  |  |  |  |  |  |  |  |  |  |  |  |  |  |  |  |  |  |  |  |  |  |  |  |  |  |  |
| Diff-sex |  |  |  |  |  |  |  |  |  |  |  |  |  |  |  |  |  |  |  |  |  |  |  |  |  |  |  |  |
| diffXTransTrip |  |  |  |  |  |  |  |  |  |  |  |  |  |  |  |  |  |  |  |  |  |  |  |  |  |  |  |  |
| **Support Networks** | 1 | 2 | 3 | 4 | 5 | 6 | 7 | 8 | 9 | 10 | 11 | 12 | 13 | 14 | 15 | 16 | 17 | 18 | 19 | 20 | 21 | 22 | 23 | 24 | 25 | 26 | 27 | 28 |
| *Rate Effects* |  |  |  |  |  |  |  |  |  |  |  |  |  |  |  |  |  |  |  |  |  |  |  |  |  |  |  |  |
| Network rate t1-t2 |  |  |  |  |  |  |  |  |  |  |  |  |  |  |  |  |  |  |  |  |  |  |  |  |  |  |  |  |
| *Structure Effects* |  |  |  |  |  |  |  |  |  |  |  |  |  |  |  |  |  |  |  |  |  |  |  |  |  |  |  |  |
| Outdegree (density) |  |  |  |  |  |  |  |  |  |  |  |  |  |  |  |  |  |  |  |  |  |  |  |  |  |  |  |  |
| Reciprocity |  |  |  |  |  |  |  |  |  |  |  |  |  |  |  |  |  |  |  |  |  |  |  |  |  |  |  |  |
| Transitive triplets |  |  |  |  |  |  |  |  |  |  |  |  |  |  |  |  |  |  |  |  |  |  |  |  |  |  |  |  |
| Indegree popularity |  |  |  |  |  |  |  |  |  |  |  |  |  |  |  |  |  |  |  |  |  |  |  |  |  |  |  |  |
| Outdegree activity |  |  |  |  |  |  |  |  |  |  |  |  |  |  |  |  |  |  |  |  |  |  |  |  |  |  |  |  |
| *Sex Effects* |  |  |  |  |  |  |  |  |  |  |  |  |  |  |  |  |  |  |  |  |  |  |  |  |  |  |  |  |
| same-sex |  |  |  |  |  |  |  |  |  |  |  |  |  |  |  |  |  |  |  |  |  |  |  |  |  |  |  |  |
| Diff-sex |  |  |  |  |  |  |  |  |  |  |  |  |  |  |  |  |  |  |  |  |  |  |  |  |  |  |  |  |
| transitive triplets different X |  |  |  |  |  |  |  |  |  |  |  |  |  |  |  |  |  |  |  |  |  |  |  |  |  |  |  |  |

*All models were analyzed using 3,000 iterations for better convergence and reliability of the parameter estimates and standard errors. In some classrooms, the estimates and standard errors of some effects were very high, and the t-ratios for convergence are very large. As a possible solution, some effects were fixed at a non-zero value (based on score-type tests) by trial and error to get better convergence (Ripley et al., 2020). Fixed parameters are indicated with black cells. Gray cells indicate effects estimated freely.*

| **Table S0B2:** Goodness of Fit statistics for SIENA multivariate network models 0-B summarized in Table 3 | | | | | | | | | |
| --- | --- | --- | --- | --- | --- | --- | --- | --- | --- |
|  | | Friendship Networks | | | | Peer Support network | | | |
| Classroom | Overall convergence | Outdegree distribution | Indegree distribution | Geodesic distance | Triad census | Outdegree distribution | Indegree distribution | Geodesic distance | Triad census |
| 1 | 0.20 | 0.31 | 0.25 | 0.24 | 0.98 | 0.76 | 0.59 | 0.33 | 0.16 |
| 2 | 0.14 | 0.23 | 0.23 | 0.46 | 0.01 | 0.24 | 0.33 | 0.34 | 0.01 |
| 3 | 0.15 | 0.52 | 0.69 | 0.37 | 0.88 | 0.05 | 0.93 | 0.03 | 0.38 |
| 4 | 0.10 | 0.94 | 0.97 | 0.74 | 1.00 | 0.83 | 0.20 | 0.94 | 0.29 |
| 5 | 0.15 | 0.06 | 0.23 | 0.83 | 0.80 | 0.28 | 0.50 | 0.21 | 0.09 |
| 6 | 0.16 | 0.17 | 0.43 | 0.58 | 0.16 | 0.81 | 0.66 | 1.00 | 0.59 |
| 7 | 0.24 | 0.11 | 1.00 | 0.94 | 0.23 | 0.72 | 0.86 | 0.86 | 0.00 |
| 8 | 0.15 | 0.13 | 0.29 | 0.99 | 0.23 | 0.35 | 0.30 | 0.74 | 0.85 |
| 9 | 0.23 | 0.00 | 0.96 | 0.27 | 0.00 | 0.49 | 0.80 | 0.53 | 0.49 |
| 10 | 0.15 | 0.21 | 0.44 | 0.18 | 0.18 | 0.35 | 0.98 | 0.79 | 1.00 |
| 11 | 0.19 | 0.57 | 0.53 | 0.94 | 0.45 | 0.07 | 0.95 | 0.25 | 0.91 |
| 12 | 0.22 | 0.10 | 0.63 | 0.11 | 0.80 | 0.04 | 0.18 | 0.60 | 0.23 |
| 13 | 0.13 | 0.04 | 0.52 | 0.97 | 0.82 | 0.12 | 0.97 | 0.27 | 0.43 |
| 14 | 0.18 | 0.42 | 0.70 | 0.63 | 1.00 | 0.90 | 0.69 | 0.84 | 0.90 |
| 15 | 0.14 | 0.08 | 0.66 | 0.84 | 0.45 | 0.07 | 0.48 | 0.71 | 0.43 |
| 16 | 0.18 | 0.54 | 0.69 | 0.61 | 0.71 | 0.28 | 0.72 | 0.59 | 0.45 |
| 17 | 0.11 | 0.27 | 0.33 | 0.79 | 0.95 | 0.48 | 0.89 | 0.97 | 0.46 |
| 18 | 0.19 | 0.00 | 0.47 | 0.97 | 0 | 0.17 | 1.00 | 0.82 | 0.00 |
| 19 | 0.22 | 0.12 | 0.92 | 0.64 | 0.43 | 0.32 | 0.88 | 0.92 | 0.71 |
| 20 | 0.18 | 0.90 | 0.01 | 0.06 | 0.44 | 0.00 | 0.62 | 0.63 | 0.77 |
| 21 | 0.14 | 0.98 | 0.66 | 0.32 | 0.88 | 0.40 | 0.15 | 0.40 | 0.51 |
| 22 | 0.16 | 0.92 | 0.96 | 0.24 | 0.01 | 0.72 | 0.08 | 0.24 | 0.80 |
| 23 | 0.11 | 0.83 | 0.36 | 0.72 | 0.94 | 0.02 | 0.87 | 0.50 | 0.29 |
| 24 | 0.12 | 0.74 | 0.92 | 0.55 | 0.61 | 0.26 | 0.83 | 0.24 | 0.10 |
| 25 | 0.19 | 0.12 | 0.09 | 0.21 | 0.24 | 0.06 | 0.15 | 0.95 | 0.18 |
| 26 | 0.19 | 0.65 | 0.62 | 0.02 | 0 | 0.92 | 0.15 | 0.33 | 0.28 |
| 27 | 0.23 | 0.37 | 0.04 | 0.06 | 0.50 | 0.43 | 0.84 | 0.95 | 0.11 |
| 28 | 0.16 | 0.14 | 0.67 | 0.51 | 0.53 | 0.09 | 0.68 | 0.90 | 0.98 |
| *Goodness of fit (GoF) was assessed with four computed auxiliary network statistics: out-degree distribution, in-degree distribution, geodesic distance, and triad census. Poor GOF (<0.05) are indicated with grey cells.* | | | | | | | | | |

**Table S0B3:** Results from RSiena analysis that test the role of sex 0-B (28 classrooms)

|  | Hypothetical change | | |  | Model 0B | | | | | | |
| --- | --- | --- | --- | --- | --- | --- | --- | --- | --- | --- | --- |
|  |  |  |  |  | Friendship network | | |  | Peer Support network | | |
|  | tx | → | tx+m |  | Est. | SE | n |  | Est. | SE | n |
| Effect parameters |  |  |  |  |  |  |  |  |  |  |  |
| Network rate |  |  |  |  | 10.90^***^ | 0.46 | 28 |  | 4.99^***^ | 0.26 | 28 |
| Structure effects |  |  |  |  |  |  |  |  |  |  |  |
| Outdegree (density) |  | **→** |  |  | -2.28^***^ | 0.12 | 28 |  | -2.82^***^ | 0.13 | 25 |
| Reciprocity |  | **→** |  |  | 1.79^***^ | 0.10 | 28 |  | 1.28^***^ | 0.08 | 27 |
| Transitive triplets |  | **→** |  |  | 0.34^***^ | 0.04 | 28 |  | 0.30^***^ | 0.03 | 28 |
| Transitive recipr. triplets |  | **→** |  |  | -0.29^***^ | 0.04 | 28 |  | —— | —— | —— |
| Number of Distance two |  | **→** |  |  | -0.18^***^ | 0.02 | 28 |  | —— | —— | —— |
| Indegree popularity |  | **→** |  |  | 0.05^***^ | 0.01 | 28 |  | 0.01 | 0.02 | 28 |
| Outdegree activity |  | **→** |  |  | 0.01 | 0.01 | 28 |  | 0.03^**^ | 0.01 | 28 |
| 4-cycles |  | **→** |  |  | -0.05^***^ | 0.01 | 28 |  | —— | —— | —— |
| Sex effects |  |  |  |  |  |  |  |  |  |  |  |
| Same-sex |  | **→** |  |  | 0.49^***^ | 0.07 | 28 |  | 1.10^***^ | 0.15 | 26 |
| Different sex |  | **→** |  |  | 0.02 | 0.06 | 28 |  | 0.12 | 0.07 | 26 |
| transitive triplets different X |  | **→** |  |  | 0.08^**^ | 0.02 | 28 |  | 0.17 | 0.37 | 20 |

*Significance was tested by dividing the estimates with the standard error, resulting in t values that were approximately normally distributed (Ripley et al., 2020). Convergence statistics: t-ratio all < 0.10, and overall maximum convergence ratio < 0.25. *p ≤ 0.05, **p ≤ 0.01, ***p ≤ 0.001 (two-tailed test).*

**Table S0B4:** Results from RSiena analysis that test the role of sex 0-B (23 classrooms (20% missing value))

|  | Hypothetical change | | |  | Model 0B | | | | | | |
| --- | --- | --- | --- | --- | --- | --- | --- | --- | --- | --- | --- |
|  |  |  |  |  | Friendship network | | |  | Peer Support network | | |
|  | tx | → | tx+m |  | Est. | SE | n |  | Est. | SE | n |
| Effect parameters |  |  |  |  |  |  |  |  |  |  |  |
| Network rate |  |  |  |  | 10.96^***^ | 0.50 | 23 |  | 4.74^***^ | 0.23 | 23 |
| Structure effects |  |  |  |  |  |  |  |  |  |  |  |
| Outdegree (density) |  | **→** |  |  | -2.23^***^ | 0.14 | 23 |  | -2.76^***^ | 0.15 | 20 |
| Reciprocity |  | **→** |  |  | 1.81^***^ | 0.12 | 23 |  | 1.31^***^ | 0.09 | 22 |
| Transitive triplets |  | **→** |  |  | 0.34^***^ | 0.05 | 23 |  | 0.33^***^ | 0.04 | 23 |
| Transitive recipr. triplets |  | **→** |  |  | -0.29^***^ | 0.05 | 23 |  | —— | —— | —— |
| Number of Distance two |  | **→** |  |  | -0.19^***^ | 0.02 | 23 |  | —— | —— | —— |
| Indegree popularity |  | **→** |  |  | 0.04^*^ | 0.01 | 23 |  | 0.00 | 0.02 | 23 |
| Outdegree activity |  | **→** |  |  | 0.00 | 0.01 | 23 |  | 0.03 | 0.01 | 23 |
| 4-cycles |  | **→** |  |  | -0.04^***^ | 0.01 | 23 |  | —— | —— | —— |
| Sex effects |  |  |  |  |  |  |  |  |  |  |  |
| Same-sex |  | **→** |  |  | 0.48^***^ | 0.01 | 23 |  | 1.18^***^ | 0.17 | 21 |
| Different sex |  | **→** |  |  | 0.05 | 0.07 | 23 |  | 012 | 0.08 | 21 |
| transitive triplets different X |  | **→** |  |  | 0.06^*^ | 0.03 | 23 |  | 0.19 | 0.49 | 13 |

*Significance was tested by dividing the estimates with the standard error, resulting in t values that were approximately normally distributed (Ripley et al., 2020). Convergence statistics: t-ratio all < 0.10, and overall maximum convergence ratio < 0.25. *p ≤ 0.05, **p ≤ 0.01, ***p ≤ 0.001 (two-tailed test).*

**Table S0B5:** Results from RSiena analysis that test the role of sex 0-B (17 classrooms, without classrooms having poor GOF)

|  | Hypothetical change | | |  | Model 0B | | | | | | |
| --- | --- | --- | --- | --- | --- | --- | --- | --- | --- | --- | --- |
|  |  |  |  |  | Friendship network | | |  | Peer Support network | | |
|  | tx | → | tx+m |  | Est. | SE | n |  | Est. | SE | n |
| Effect parameters |  |  |  |  |  |  |  |  |  |  |  |
| Network rate |  |  |  |  | 10.40^***^ | 0.58 | 16 |  | 4.99^***^ | 0.34 | 16 |
| Structure effects |  |  |  |  |  |  |  |  |  |  |  |
| Outdegree (density) |  | **→** |  |  | -2.23^***^ | 0.16 | 16 |  | -2.94^***^ | 0.17 | 15 |
| Reciprocity |  | **→** |  |  | 1.80^***^ | 0.14 | 16 |  | 1.13^***^ | 0.11 | 15 |
| Transitive triplets |  | **→** |  |  | 0.34^***^ | 0.04 | 16 |  | 0.27^**^ | 0.05 | 16 |
| Transitive recipr. triplets |  | **→** |  |  | -0.33^***^ | 0.05 | 16 |  | —— | —— | —— |
| Number of Distance two |  | **→** |  |  | -0.18^***^ | 0.03 | 16 |  | —— | —— | —— |
| Indegree popularity |  | **→** |  |  | 0.05^*^ | 0.02 | 16 |  | 0.06^*^ | 0.02 | 16 |
| Outdegree activity |  | **→** |  |  | 0.01 | 0.01 | 16 |  | 0.06^***^ | 0.01 | 14 |
| 4-cycles |  | **→** |  |  | -0.03^**^ | 0.01 | 16 |  | —— | —— | —— |
| Sex effects |  |  |  |  |  |  |  |  |  |  |  |
| Same-sex |  | **→** |  |  | 0.51^***^ | 0.10 | 16 |  | 0.97^***^ | 0.16 | 16 |
| Different sex |  | **→** |  |  | -0.05 | 0.07 | 16 |  | 0.08 | 0.09 | 16 |
| transitive triplets different X |  | **→** |  |  | 0.09^*^ | 0.04 | 16 |  | 0.25 | 0.12 | 12 |

*Significance was tested by dividing the estimates with the standard error, resulting in t values that were approximately normally distributed (Ripley et al., 2020). Convergence statistics: t-ratio all < 0.10, and overall maximum convergence ratio < 0.25. *p ≤ 0.05, **p ≤ 0.01, ***p ≤ 0.001 (two-tailed test).*

**Table S0C1:** Effects included in all SIENA multivariate network models 0-C summarized in Table 3

| **Friendship Networks** | 1 | 2 | 3 | 4 | 5 | 6 | 7 | 8 | 9 | 10 | 11 | 12 | 13 | 14 | 15 | 16 | 17 | 18 | 19 | 20 | 21 | 22 | 23 | 24 | 25 | 26 | 27 | 28 |
| --- | --- | --- | --- | --- | --- | --- | --- | --- | --- | --- | --- | --- | --- | --- | --- | --- | --- | --- | --- | --- | --- | --- | --- | --- | --- | --- | --- | --- |
| *Rate Effects* |  |  |  |  |  |  |  |  |  |  |  |  |  |  |  |  |  |  |  |  |  |  |  |  |  |  |  |  |
| Network rate t1-t2 |  |  |  |  |  |  |  |  |  |  |  |  |  |  |  |  |  |  |  |  |  |  |  |  |  |  |  |  |
| *Structure Effects* |  |  |  |  |  |  |  |  |  |  |  |  |  |  |  |  |  |  |  |  |  |  |  |  |  |  |  |  |
| Outdegree (density) |  |  |  |  |  |  |  |  |  |  |  |  |  |  |  |  |  |  |  |  |  |  |  |  |  |  |  |  |
| Reciprocity |  |  |  |  |  |  |  |  |  |  |  |  |  |  |  |  |  |  |  |  |  |  |  |  |  |  |  |  |
| Transitive triplets |  |  |  |  |  |  |  |  |  |  |  |  |  |  |  |  |  |  |  |  |  |  |  |  |  |  |  |  |
| Transitive reciprocated triplets |  |  |  |  |  |  |  |  |  |  |  |  |  |  |  |  |  |  |  |  |  |  |  |  |  |  |  |  |
| Number of distance 2 |  |  |  |  |  |  |  |  |  |  |  |  |  |  |  |  |  |  |  |  |  |  |  |  |  |  |  |  |
| Indegree popularity |  |  |  |  |  |  |  |  |  |  |  |  |  |  |  |  |  |  |  |  |  |  |  |  |  |  |  |  |
| Outdegree activity |  |  |  |  |  |  |  |  |  |  |  |  |  |  |  |  |  |  |  |  |  |  |  |  |  |  |  |  |
| Four-cycles |  |  |  |  |  |  |  |  |  |  |  |  |  |  |  |  |  |  |  |  |  |  |  |  |  |  |  |  |
| *Sex Effects* |  |  |  |  |  |  |  |  |  |  |  |  |  |  |  |  |  |  |  |  |  |  |  |  |  |  |  |  |
| Same-sex |  |  |  |  |  |  |  |  |  |  |  |  |  |  |  |  |  |  |  |  |  |  |  |  |  |  |  |  |
| Diff sex |  |  |  |  |  |  |  |  |  |  |  |  |  |  |  |  |  |  |  |  |  |  |  |  |  |  |  |  |
| homXTransTrip |  |  |  |  |  |  |  |  |  |  |  |  |  |  |  |  |  |  |  |  |  |  |  |  |  |  |  |  |
| **Support Networks** | 1 | 2 | 3 | 4 | 5 | 6 | 7 | 8 | 9 | 10 | 11 | 12 | 13 | 14 | 15 | 16 | 17 | 18 | 19 | 20 | 21 | 22 | 23 | 24 | 25 | 26 | 27 | 28 |
| *Rate Effects* |  |  |  |  |  |  |  |  |  |  |  |  |  |  |  |  |  |  |  |  |  |  |  |  |  |  |  |  |
| Network rate t1-t2 |  |  |  |  |  |  |  |  |  |  |  |  |  |  |  |  |  |  |  |  |  |  |  |  |  |  |  |  |
| *Structure Effects* |  |  |  |  |  |  |  |  |  |  |  |  |  |  |  |  |  |  |  |  |  |  |  |  |  |  |  |  |
| Outdegree (density) |  |  |  |  |  |  |  |  |  |  |  |  |  |  |  |  |  |  |  |  |  |  |  |  |  |  |  |  |
| Reciprocity |  |  |  |  |  |  |  |  |  |  |  |  |  |  |  |  |  |  |  |  |  |  |  |  |  |  |  |  |
| Transitive triplets |  |  |  |  |  |  |  |  |  |  |  |  |  |  |  |  |  |  |  |  |  |  |  |  |  |  |  |  |
| Indegree popularity |  |  |  |  |  |  |  |  |  |  |  |  |  |  |  |  |  |  |  |  |  |  |  |  |  |  |  |  |
| Outdegree activity |  |  |  |  |  |  |  |  |  |  |  |  |  |  |  |  |  |  |  |  |  |  |  |  |  |  |  |  |
| *Sex Effects* |  |  |  |  |  |  |  |  |  |  |  |  |  |  |  |  |  |  |  |  |  |  |  |  |  |  |  |  |
| Same-sex |  |  |  |  |  |  |  |  |  |  |  |  |  |  |  |  |  |  |  |  |  |  |  |  |  |  |  |  |
| Diff-sex |  |  |  |  |  |  |  |  |  |  |  |  |  |  |  |  |  |  |  |  |  |  |  |  |  |  |  |  |
| transitive triplets homogeneous X |  |  |  |  |  |  |  |  |  |  |  |  |  |  |  |  |  |  |  |  |  |  |  |  |  |  |  |  |

*All models were analyzed using 3,000 iterations for better convergence and reliability of the parameter estimates and standard errors. In some classrooms, the estimates and standard errors of some effects were very high, and the t-ratios for convergence are very large. As a possible solution, some effects were fixed at a non-zero value (based on score-type tests) by trial and error to get better convergence (Ripley et al., 2020). Fixed parameters are indicated with black cells. Gray cells indicate effects estimated freely. Because the models of two classrooms can not get convergence (classrooms 13 & 15), the two classrooms were not included in model 0-C.*

| **Table S0C2:** Goodness of Fit statistics for SIENA multivariate network models 0-C summarized in Table 3 | | | | | | | | | |
| --- | --- | --- | --- | --- | --- | --- | --- | --- | --- |
|  | | Friendship Networks | | | | Peer Support network | | | |
| Classroom | Overall convergence | Outdegree distribution | Indegree distribution | Geodesic distance | Triad census | Outdegree distribution | Indegree distribution | Geodesic distance | Triad census |
| 1 | 0.11 | 0.50 | 0.12 | 0.13 | 0.54 | 0.68 | 0.54 | 0.42 | 0.41 |
| 2 | 0.16 | 0.18 | 0.25 | 0.46 | 0.01 | 0.29 | 0.35 | 0.33 | 0.02 |
| 3 | 0.18 | 0.55 | 0.76 | 0.41 | 0.96 | 0.05 | 0.91 | 0.03 | 0.53 |
| 4 | 0.18 | 0.93 | 0.95 | 0.71 | 1.00 | 0.80 | 0.21 | 0.94 | 0.44 |
| 5 | 0.15 | 0.05 | 0.25 | 0.80 | 0.75 | 0.31 | 0.24 | 0.28 | 0.48 |
| 6 | 0.23 | 0.16 | 0.40 | 0.57 | 0.16 | 0.85 | 0.64 | 0.99 | 0.43 |
| 7 | 0.18 | 0.08 | 0.99 | 0.92 | 0.03 | 0.74 | 0.87 | 0.84 | 0.01 |
| 8 | 0.15 | 0.08 | 0.34 | 0.99 | 0.27 | 0.34 | 0.39 | 0.76 | 0.89 |
| 9 | 0.22 | 0.01 | 0.99 | 0.25 | 0.00 | 0.48 | 0.74 | 0.49 | 0.46 |
| 10 | 0.18 | 0.35 | 0.48 | 0.20 | 0.23 | 0.39 | 0.97 | 0.81 | 1.00 |
| 11 | 0.18 | 0.63 | 0.50 | 0.83 | 0.47 | 0.08 | 0.98 | 0.28 | 0.89 |
| 12 | 0.15 | 0.12 | 0.57 | 0.09 | 0.93 | 0.06 | 0.09 | 0.21 | 0.32 |
| 13 |  |  |  |  |  |  |  |  |  |
| 14 | 0.16 | 0.38 | 0.68 | 0.60 | 1.00 | 0.90 | 0.68 | 0.82 | 0.96 |
| 15 |  |  |  |  |  |  |  |  |  |
| 16 | 0.17 | 0.48 | 0.50 | 0.56 | 0.49 | 0.24 | 0.76 | 0.61 | 0.67 |
| 17 | 0.17 | 0.27 | 0.38 | 0.83 | 0.97 | 0.49 | 0.85 | 0.98 | 0.57 |
| 18 | 0.22 | 0.00 | 0.52 | 0.94 | 0 | 0.10 | 0.98 | 0.87 | 0 |
| 19 | 0.23 | 0.15 | 0.94 | 0.58 | 0.28 | 0.35 | 0.87 | 0.93 | 0.82 |
| 20 | 0.16 | 0.91 | 0.02 | 0.06 | 0.46 | 0 | 0.64 | 0.68 | 0.79 |
| 21 | 0.15 | 0.93 | 0.21 | 0.46 | 0.98 | 0.42 | 0.16 | 0.42 | 0.5 |
| 22 | 0.12 | 0.91 | 0.95 | 0.22 | 0.00 | 0.78 | 0.10 | 0.26 | 0.74 |
| 23 | 0.16 | 0.84 | 0.36 | 0.70 | 0.72 | 0.16 | 0.65 | 0.12 | 0.10 |
| 24 | 0.18 | 0.71 | 0.90 | 0.53 | 0.74 | 0.23 | 0.90 | 0.87 | 0.04 |
| 25 | 0.23 | 0.19 | 0.13 | 0.21 | 0.16 | 0.05 | 0.10 | 0.96 | 0.15 |
| 26 | 0.16 | 0.63 | 0.61 | 0.01 | 0 | 0.93 | 0.14 | 0.36 | 0.38 |
| 27 | 0.13 | 0.30 | 0.04 | 0.07 | 0.44 | 0.42 | 0.87 | 0.98 | 0.25 |
| 28 | 0.19 | 0.07 | 0.68 | 0.52 | 0.42 | 0.12 | 0.68 | 0.91 | 0.98 |
| *Goodness of fit (GoF) was assessed with four computed auxiliary network statistics: out-degree distribution, in-degree distribution, geodesic distance, and triad census. Poor GOF (<0.05) are indicated with grey cells.* | | | | | | | | | |

**Table S0C3:** Results from RSiena analysis that test the role of sex 0-C (26 classrooms)

|  | Hypothetical change | | |  | Model 0C | | | | | | |
| --- | --- | --- | --- | --- | --- | --- | --- | --- | --- | --- | --- |
|  |  |  |  |  | Friendship network | | |  | Peer Support network | | |
|  | tx | → | tx+m |  | Est. | SE | n |  | Est. | SE | n |
| Effect parameters |  |  |  |  |  |  |  |  |  |  |  |
| Network rate |  |  |  |  | 10.67^***^ | 0.47 | 26 |  | 4.74^***^ | 0.27 | 26 |
| Structure effects |  |  |  |  |  |  |  |  |  |  |  |
| Outdegree (density) |  | **→** |  |  | -2.32^***^ | 0.13 | 25 |  | -2.81^***^ | 0.16 | 22 |
| Reciprocity |  | **→** |  |  | 1.72^***^ | 0.11 | 25 |  | 1.43^***^ | 0.10 | 26 |
| Transitive triplets |  | **→** |  |  | 0.45^***^ | 0.05 | 24 |  | 0.58^***^ | 0.17 | 16 |
| Transitive recipr. triplets |  | **→** |  |  | -0.25^***^ | 0.04 | 26 |  | —— | —— | —— |
| Number of Distance two |  | **→** |  |  | -0.17^***^ | 0.02 | 26 |  | —— | —— | —— |
| Indegree popularity |  | **→** |  |  | 0.05^***^ | 0.01 | 26 |  | 0.03 | 0.02 | 26 |
| Outdegree activity |  | **→** |  |  | 0.02 | 0.01 | 26 |  | 0.04^**^ | 0.01 | 26 |
| 4-cycles |  | **→** |  |  | -0.05^***^ | 0.01 | 26 |  | —— | —— | —— |
| Sex effects |  |  |  |  |  |  |  |  |  |  |  |
| Same-sex |  | **→** |  |  | 0.47^***^ | 0.07 | 25 |  | 0.80^***^ | 0.11 | 25 |
| Different sex |  | **→** |  |  | 0.04 | 0.07 | 26 |  | 0.12 | 0.08 | 24 |
| transitive triplets homogeneous X |  | **→** |  |  | -0.20^**^ | 0.06 | 26 |  | 0.52 | 0.45 | 23 |

*Significance was tested by dividing the estimates with the standard error, resulting in t values that were approximately normally distributed (Ripley et al., 2020). Convergence statistics: t-ratio all < 0.10, and overall maximum convergence ratio < 0.25. *p ≤ 0.05, **p ≤ 0.01, ***p ≤ 0.001 (two-tailed test).*

**Table S0C4:** Results from RSiena analysis that test the role of sex 0-C (21 classrooms (20% missing value))

|  | Hypothetical change | | |  | Model 0C | | | | | | |
| --- | --- | --- | --- | --- | --- | --- | --- | --- | --- | --- | --- |
|  |  |  |  |  | Friendship network | | |  | Peer Support network | | |
|  | tx | → | tx+m |  | Est. | SE | n |  | Est. | SE | n |
| Effect parameters |  |  |  |  |  |  |  |  |  |  |  |
| Network rate |  |  |  |  | 10.68^***^ | 0.50 | 21 |  | 4.42^***^ | 0.23 | 21 |
| Structure effects |  |  |  |  |  |  |  |  |  |  |  |
| Outdegree (density) |  | **→** |  |  | -2.29^***^ | 0.14 | 20 |  | -2.71^***^ | 0.19 | 17 |
| Reciprocity |  | **→** |  |  | 1.74^***^ | 0.12 | 20 |  | 1.51^***^ | 0.12 | 21 |
| Transitive triplets |  | **→** |  |  | 0.44^***^ | 0.06 | 19 |  | 0.75^***^ | 0.26 | 12 |
| Transitive recipr. triplets |  | **→** |  |  | -0.26^***^ | 0.05 | 21 |  | —— | —— | —— |
| Number of Distance two |  | **→** |  |  | -0.17^***^ | 0.02 | 21 |  | —— | —— | —— |
| Indegree popularity |  | **→** |  |  | 0.04^*^ | 0.02 | 21 |  | 0.01 | 0.03 | 21 |
| Outdegree activity |  | **→** |  |  | 0.01 | 0.01 | 21 |  | 0.04^*^ | 0.02 | 21 |
| 4-cycles |  | **→** |  |  | -0.04^***^ | 0.01 | 21 |  | —— | —— | —— |
| Sex effects |  |  |  |  |  |  |  |  |  |  |  |
| Same-sex |  | **→** |  |  | 0.45^***^ | 0.07 | 20 |  | 0.82^***^ | 0.14 | 20 |
| Different sex |  | **→** |  |  | 0.04 | 0.09 | 21 |  | 0.11 | 0.10 | 19 |
| transitive triplets homogeneous X |  | **→** |  |  | -0.14 | 0.07 | 21 |  | 0.64 | 0.58 | 18 |

*Significance was tested by dividing the estimates with the standard error, resulting in t values that were approximately normally distributed (Ripley et al., 2020). Convergence statistics: t-ratio all < 0.10, and overall maximum convergence ratio < 0.25. *p ≤ 0.05, **p ≤ 0.01, ***p ≤ 0.001 (two-tailed test).*

**Table S0C5:** Results from RSiena analysis that test the role of sex 0-C (16 classrooms, without classrooms having poor GOF)

|  | Hypothetical change | | |  | Model 0C | | | | | | |
| --- | --- | --- | --- | --- | --- | --- | --- | --- | --- | --- | --- |
|  |  |  |  |  | Friendship network | | |  | Peer Support network | | |
|  | tx | → | tx+m |  | Est. | SE | n |  | Est. | SE | n |
| Effect parameters |  |  |  |  |  |  |  |  |  |  |  |
| Network rate |  |  |  |  | 10.26^***^ | 0.60 | 16 |  | 4.97^***^ | 0.38 | 16 |
| Structure effects |  |  |  |  |  |  |  |  |  |  |  |
| Outdegree (density) |  | **→** |  |  | -2.45^***^ | 0.17 | 15 |  | -2.97^***^ | 0.21 | 13 |
| Reciprocity |  | **→** |  |  | 1.62^***^ | 0.15 | 15 |  | 1.27^***^ | 0.14 | 16 |
| Transitive triplets |  | **→** |  |  | 0.51^***^ | 0.06 | 14 |  | 0.29 | 0.16 | 10 |
| Transitive recipr. triplets |  | **→** |  |  | -0.36^***^ | 0.05 | 15 |  | —— | —— | —— |
| Number of Distance two |  | **→** |  |  | -0.16^***^ | 0.03 | 16 |  | —— | —— | —— |
| Indegree popularity |  | **→** |  |  | 0.06^*^ | 0.02 | 16 |  | 0.07^*^ | 0.02 | 16 |
| Outdegree activity |  | **→** |  |  | 0.03^**^ | 0.01 | 15 |  | 0.06^***^ | 0.01 | 15 |
| 4-cycles |  | **→** |  |  | -0.04^***^ | 0.01 | 16 |  | —— | —— | —— |
| Sex effects |  |  |  |  |  |  |  |  |  |  |  |
| Same-sex |  | **→** |  |  | 0.53^***^ | 0.10 | 15 |  | 0.84^***^ | 0.13 | 15 |
| Different sex |  | **→** |  |  | -0.05 | 0.08 | 16 |  | 0.11 | 0.10 | 15 |
| transitive triplets homogeneous X |  | **→** |  |  | -0.27^**^ | 0.08 | 16 |  | 0.42 | 0.36 | 14 |

*Significance was tested by dividing the estimates with the standard error, resulting in t values that were approximately normally distributed (Ripley et al., 2020). Convergence statistics: t-ratio all < 0.10, and overall maximum convergence ratio < 0.25. *p ≤ 0.05, **p ≤ 0.01, ***p ≤ 0.001 (two-tailed test).*

**Table S0D1:** Effects included in all SIENA multivariate network models 0-D summarized in Table 3

| **Friendship Networks** | 1 | 2 | 3 | 4 | 5 | 6 | 7 | 8 | 9 | 10 | 11 | 12 | 13 | 14 | 15 | 16 | 17 | 18 | 19 | 20 | 21 | 22 | 23 | 24 | 25 | 26 | 27 | 28 |
| --- | --- | --- | --- | --- | --- | --- | --- | --- | --- | --- | --- | --- | --- | --- | --- | --- | --- | --- | --- | --- | --- | --- | --- | --- | --- | --- | --- | --- |
| *Rate Effects* |  |  |  |  |  |  |  |  |  |  |  |  |  |  |  |  |  |  |  |  |  |  |  |  |  |  |  |  |
| Network rate t1-t2 |  |  |  |  |  |  |  |  |  |  |  |  |  |  |  |  |  |  |  |  |  |  |  |  |  |  |  |  |
| *Structure Effects* |  |  |  |  |  |  |  |  |  |  |  |  |  |  |  |  |  |  |  |  |  |  |  |  |  |  |  |  |
| Outdegree (density) |  |  |  |  |  |  |  |  |  |  |  |  |  |  |  |  |  |  |  |  |  |  |  |  |  |  |  |  |
| Reciprocity |  |  |  |  |  |  |  |  |  |  |  |  |  |  |  |  |  |  |  |  |  |  |  |  |  |  |  |  |
| Transitive triplets |  |  |  |  |  |  |  |  |  |  |  |  |  |  |  |  |  |  |  |  |  |  |  |  |  |  |  |  |
| Transitive reciprocated triplets |  |  |  |  |  |  |  |  |  |  |  |  |  |  |  |  |  |  |  |  |  |  |  |  |  |  |  |  |
| Number of distance 2 |  |  |  |  |  |  |  |  |  |  |  |  |  |  |  |  |  |  |  |  |  |  |  |  |  |  |  |  |
| Indegree popularity |  |  |  |  |  |  |  |  |  |  |  |  |  |  |  |  |  |  |  |  |  |  |  |  |  |  |  |  |
| Outdegree activity |  |  |  |  |  |  |  |  |  |  |  |  |  |  |  |  |  |  |  |  |  |  |  |  |  |  |  |  |
| Four-cycles |  |  |  |  |  |  |  |  |  |  |  |  |  |  |  |  |  |  |  |  |  |  |  |  |  |  |  |  |
| *Sex Effects* |  |  |  |  |  |  |  |  |  |  |  |  |  |  |  |  |  |  |  |  |  |  |  |  |  |  |  |  |
| Same-sex |  |  |  |  |  |  |  |  |  |  |  |  |  |  |  |  |  |  |  |  |  |  |  |  |  |  |  |  |
| Diff-sex |  |  |  |  |  |  |  |  |  |  |  |  |  |  |  |  |  |  |  |  |  |  |  |  |  |  |  |  |
| jumpXTransTrip |  |  |  |  |  |  |  |  |  |  |  |  |  |  |  |  |  |  |  |  |  |  |  |  |  |  |  |  |
| **Support Networks** | 1 | 2 | 3 | 4 | 5 | 6 | 7 | 8 | 9 | 10 | 11 | 12 | 13 | 14 | 15 | 16 | 17 | 18 | 19 | 20 | 21 | 22 | 23 | 24 | 25 | 26 | 27 | 28 |
| *Rate Effects* |  |  |  |  |  |  |  |  |  |  |  |  |  |  |  |  |  |  |  |  |  |  |  |  |  |  |  |  |
| Network rate t1-t2 |  |  |  |  |  |  |  |  |  |  |  |  |  |  |  |  |  |  |  |  |  |  |  |  |  |  |  |  |
| *Structure Effects* |  |  |  |  |  |  |  |  |  |  |  |  |  |  |  |  |  |  |  |  |  |  |  |  |  |  |  |  |
| Outdegree (density) |  |  |  |  |  |  |  |  |  |  |  |  |  |  |  |  |  |  |  |  |  |  |  |  |  |  |  |  |
| Reciprocity |  |  |  |  |  |  |  |  |  |  |  |  |  |  |  |  |  |  |  |  |  |  |  |  |  |  |  |  |
| Transitive triplets |  |  |  |  |  |  |  |  |  |  |  |  |  |  |  |  |  |  |  |  |  |  |  |  |  |  |  |  |
| Indegree popularity |  |  |  |  |  |  |  |  |  |  |  |  |  |  |  |  |  |  |  |  |  |  |  |  |  |  |  |  |
| Outdegree activity |  |  |  |  |  |  |  |  |  |  |  |  |  |  |  |  |  |  |  |  |  |  |  |  |  |  |  |  |
| *Sex Effects* |  |  |  |  |  |  |  |  |  |  |  |  |  |  |  |  |  |  |  |  |  |  |  |  |  |  |  |  |
| same-sex |  |  |  |  |  |  |  |  |  |  |  |  |  |  |  |  |  |  |  |  |  |  |  |  |  |  |  |  |
| Diff-sex |  |  |  |  |  |  |  |  |  |  |  |  |  |  |  |  |  |  |  |  |  |  |  |  |  |  |  |  |
| transitive triplets jumping to different X |  |  |  |  |  |  |  |  |  |  |  |  |  |  |  |  |  |  |  |  |  |  |  |  |  |  |  |  |

*All models were analyzed using 3,000 iterations for better convergence and reliability of the parameter estimates and standard errors. In some classrooms, the estimates and standard errors of some effects were very high, and the t-ratios for convergence are very large. As a possible solution, some effects were fixed at a non-zero value (based on score-type tests) by trial and error to get better convergence (Ripley et al., 2020). Fixed parameters are indicated with black cells. Gray cells indicate effects estimated freely.*

| **Table S0D2:** Goodness of Fit statistics for SIENA multivariate network models 0-D summarized in Table 3 | | | | | | | | | |
| --- | --- | --- | --- | --- | --- | --- | --- | --- | --- |
|  | | Friendship Networks | | | | Peer Support network | | | |
| Classroom | Overall convergence | Outdegree distribution | Indegree distribution | Geodesic distance | Triad census | Outdegree distribution | Indegree distribution | Geodesic distance | Triad census |
| 1 | 0.14 | 0.30 | 0.26 | 0.21 | 1.00 | 0.77 | 0.59 | 0.32 | 0.16 |
| 2 | 0.17 | 0.08 | 0.18 | 0.26 | 0.00 | 0.28 | 0.35 | 0.34 | 0.02 |
| 3 | 0.19 | 0.52 | 0.66 | 0.30 | 0.87 | 0.06 | 0.89 | 0.03 | 0.45 |
| 4 | 0.19 | 0.95 | 0.96 | 0.76 | 1 | 0.79 | 0.20 | 0.95 | 0.23 |
| 5 | 0.14 | 0.09 | 0.25 | 0.77 | 0.80 | 0.25 | 0.49 | 0.1 | 0.02 |
| 6 | 0.20 | 0.18 | 0.38 | 0.64 | 0.12 | 0.80 | 0.72 | 1.00 | 0.55 |
| 7 | 0.22 | 0.11 | 0.99 | 0.95 | 0.22 | 0.72 | 0.87 | 0.76 | 0 |
| 8 | 0.21 | 0.08 | 0.37 | 0.99 | 0.28 | 0.35 | 0.30 | 0.71 | 0.88 |
| 9 | 0.16 | 0.01 | 0.99 | 0.28 | 0.00 | 0.55 | 0.81 | 0.53 | 0.56 |
| 10 | 0.11 | 0.15 | 0.42 | 0.10 | 0.10 | 0.42 | 0.98 | 0.82 | 0.99 |
| 11 | 0.16 | 0.51 | 0.47 | 0.95 | 0.45 | 0.08 | 0.97 | 0.27 | 0.94 |
| 12 | 0.24 | 0.12 | 0.59 | 0.11 | 0.83 | 0.03 | 0.15 | 0.56 | 0.25 |
| 13 | 0.21 | 0.01 | 0.37 | 1.00 | 0.96 | 0.11 | 0.95 | 0.28 | 0.43 |
| 14 | 0.15 | 0.39 | 0.67 | 0.66 | 1 | 0.90 | 0.70 | 0.87 | 0.89 |
| 15 | 0.17 | 0.09 | 0.65 | 0.81 | 0.53 | 0.09 | 0.38 | 0.69 | 0.47 |
| 16 | 0.16 | 0.16 | 0.63 | 0.56 | 0.36 | 0.31 | 0.73 | 0.54 | 0.42 |
| 17 | 0.11 | 0.29 | 0.29 | 0.89 | 0.97 | 0.40 | 0.88 | 0.94 | 0.31 |
| 18 | 0.24 | 0.00 | 0.44 | 1.00 | 0 | 0.13 | 1.00 | 0.90 | 0.00 |
| 19 | 0.24 | 0.08 | 0.91 | 0.57 | 0.44 | 0.34 | 0.88 | 0.91 | 0.78 |
| 20 | 0.17 | 0.85 | 0.02 | 0.08 | 0.66 | 0.00 | 0.76 | 0.83 | 0.97 |
| 21 | 0.17 | 0.99 | 0.69 | 0.37 | 0.87 | 0.42 | 0.18 | 0.35 | 0.44 |
| 22 | 0.16 | 0.88 | 0.96 | 0.26 | 0.01 | 0.74 | 0.10 | 0.27 | 0.65 |
| 23 | 0.17 | 0.83 | 0.37 | 0.62 | 0.97 | 0.03 | 0.85 | 0.54 | 0.21 |
| 24 | 0.20 | 0.72 | 0.91 | 0.60 | 0.73 | 0.26 | 0.86 | 0.25 | 0.09 |
| 25 | 0.20 | 0.17 | 0.11 | 0.15 | 0.27 | 0.07 | 0.12 | 0.95 | 0.20 |
| 26 | 0.15 | 0.62 | 0.62 | 0.02 | 0 | 0.90 | 0.15 | 0.33 | 0.15 |
| 27 | 0.14 | 0.33 | 0.03 | 0.06 | 0.56 | 0.56 | 0.83 | 0.88 | 0.06 |
| 28 | 0.16 | 0.09 | 0.68 | 0.51 | 0.51 | 0.11 | 0.65 | 0.91 | 0.98 |
| *Goodness of fit (GoF) was assessed with four computed auxiliary network statistics: out-degree distribution, in-degree distribution, geodesic distance, and triad census. Poor GOF (<0.05) are indicated with grey cells.* | | | | | | | | | |

**Table S0D3:** Results from RSiena analysis that test the role of sex 0-D (28 classrooms)

|  | Hypothetical change | | |  | Model 0D | | | | | | |
| --- | --- | --- | --- | --- | --- | --- | --- | --- | --- | --- | --- |
|  |  |  |  |  | Friendship network | | |  | Peer Support network | | |
|  | tx | → | tx+m |  | Est. | SE | n |  | Est. | SE | n |
| Effect parameters |  |  |  |  |  |  |  |  |  |  |  |
| Network rate |  |  |  |  | 10.83^***^ | 0.44 | 28 |  | 5.11^***^ | 0.28 | 28 |
| Structure effects |  |  |  |  |  |  |  |  |  |  |  |
| Outdegree (density) |  | **→** |  |  | -2.27^***^ | 0.13 | 28 |  | -2.76^***^ | 0.13 | 27 |
| Reciprocity |  | **→** |  |  | 1.85^***^ | 0.09 | 28 |  | 1.28^***^ | 0.08 | 27 |
| Transitive triplets |  | **→** |  |  | 0.37^***^ | 0.04 | 28 |  | 0.32^***^ | 0.04 | 28 |
| Transitive recipr. triplets |  | **→** |  |  | -0.32^***^ | 0.04 | 28 |  | —— | —— | —— |
| Number of Distance two |  | **→** |  |  | -0.19^***^ | 0.02 | 28 |  | —— | —— | —— |
| Indegree popularity |  | **→** |  |  | 0.05^***^ | 0.01 | 28 |  | 0.02 | 0.02 | 28 |
| Outdegree activity |  | **→** |  |  | 0.01 | 0.01 | 28 |  | 0.04^**^ | 0.01 | 28 |
| 4-cycles |  | **→** |  |  | -0.05^***^ | 0.01 | 28 |  | —— | —— | —— |
| Sex effects |  |  |  |  |  |  |  |  |  |  |  |
| Same-sex |  | **→** |  |  | 0.34^***^ | 0.07 | 28 |  | 0.78^***^ | 0.10 | 27 |
| Different sex |  | **→** |  |  | 0.06 | 0.06 | 28 |  | 0.12 | 0.07 | 25 |
| transitive triplets jumping to  different X |  | **→** |  |  | -0.08 | 0.06 | 28 |  | -0.59 | 0.61 | 17 |

*Significance was tested by dividing the estimates with the standard error, resulting in t values that were approximately normally distributed (Ripley et al., 2020). Convergence statistics: t-ratio all < 0.10, and overall maximum convergence ratio < 0.25. *p ≤ 0.05, **p ≤ 0.01, ***p ≤ 0.001 (two-tailed test).*

**Table S0D4:** Results from RSiena analysis that test the role of sex 0-D (23 classrooms (20% missing value))

|  | Hypothetical change | | |  | Model 0D | | | | | | |
| --- | --- | --- | --- | --- | --- | --- | --- | --- | --- | --- | --- |
|  |  |  |  |  | Friendship network | | |  | Peer Support network | | |
|  | tx | → | tx+m |  | Est. | SE | n |  | Est. | SE | n |
| Effect parameters |  |  |  |  |  |  |  |  |  |  |  |
| Network rate |  |  |  |  | 10.80^***^ | 0.50 | 23 |  | 4.83^***^ | 0.24 | 23 |
| Structure effects |  |  |  |  |  |  |  |  |  |  |  |
| Outdegree (density) |  | **→** |  |  | -2.21^***^ | 0.14 | 23 |  | -2.70^***^ | 0.14 | 22 |
| Reciprocity |  | **→** |  |  | 1.86^***^ | 0.10 | 23 |  | 1.30^***^ | 0.09 | 23 |
| Transitive triplets |  | **→** |  |  | 0.37^***^ | 0.05 | 23 |  | 0.36^***^ | 0.04 | 23 |
| Transitive recipr. triplets |  | **→** |  |  | -0.31^***^ | 0.05 | 23 |  | —— | —— | —— |
| Number of Distance two |  | **→** |  |  | -0.19^***^ | 0.02 | 23 |  | —— | —— | —— |
| Indegree popularity |  | **→** |  |  | 0.04^**^ | 0.01 | 23 |  | 0.00 | 0.02 | 23 |
| Outdegree activity |  | **→** |  |  | 0.00 | 0.01 | 23 |  | 0.04^*^ | 0.01 | 23 |
| 4-cycles |  | **→** |  |  | -0.04^***^ | 0.01 | 23 |  | —— | —— | —— |
| Sex effects |  |  |  |  |  |  |  |  |  |  |  |
| Same-sex |  | **→** |  |  | 0.34^***^ | 0.07 | 23 |  | 0.79^***^ | 0.12 | 22 |
| Different sex |  | **→** |  |  | 0.08 | 0.07 | 23 |  | 0.12 | 0.08 | 21 |
| transitive triplets jumping to  different X |  | **→** |  |  | -0.11^*^ | 0.07 | 21 |  | -0.95 | 0.86 | 12 |

*Significance was tested by dividing the estimates with the standard error, resulting in t values that were approximately normally distributed (Ripley et al., 2020). Convergence statistics: t-ratio all < 0.10, and overall maximum convergence ratio < 0.25. *p ≤ 0.05, **p ≤ 0.01, ***p ≤ 0.001 (two-tailed test).*

**Table S0D5:** Results from RSiena analysis that test the role of sex 0-D (17 classrooms, without classrooms having poor GOF)

|  | Hypothetical change | | |  | Model 0D | | | | | | |
| --- | --- | --- | --- | --- | --- | --- | --- | --- | --- | --- | --- |
|  |  |  |  |  | Friendship network | | |  | Peer Support network | | |
|  | tx | → | tx+m |  | Est. | SE | n |  | Est. | SE | n |
| Effect parameters |  |  |  |  |  |  |  |  |  |  |  |
| Network rate |  |  |  |  | 10.66^***^ | 0.59 | 15 |  | 5.20^***^ | 0.36 | 15 |
| Structure effects |  |  |  |  |  |  |  |  |  |  |  |
| Outdegree (density) |  | **→** |  |  | -2.25^***^ | 0.18 | 15 |  | -2.87^***^ | 0.17 | 15 |
| Reciprocity |  | **→** |  |  | 1.83^***^ | 0.14 | 15 |  | 1.15^***^ | 0.11 | 15 |
| Transitive triplets |  | **→** |  |  | 0.37^***^ | 0.04 | 15 |  | 0.31^**^ | 0.06 | 15 |
| Transitive recipr. triplets |  | **→** |  |  | -0.36^***^ | 0.05 | 15 |  | —— | —— | —— |
| Number of Distance two |  | **→** |  |  | -0.20^***^ | 0.03 | 15 |  | —— | —— | —— |
| Indegree popularity |  | **→** |  |  | 0.05^*^ | 0.02 | 15 |  | 0.07^*^ | 0.02 | 15 |
| Outdegree activity |  | **→** |  |  | 0.02 | 0.01 | 15 |  | 0.06^***^ | 0.01 | 15 |
| 4-cycles |  | **→** |  |  | -0.03^**^ | 0.01 | 15 |  | —— | —— | —— |
| Sex effects |  |  |  |  |  |  |  |  |  |  |  |
| Same-sex |  | **→** |  |  | 0.37^***^ | 0.09 | 15 |  | 0.70^***^ | 0.10 | 15 |
| Different sex |  | **→** |  |  | -0.01 | 0.09 | 15 |  | 0.08 | 0.09 | 14 |
| transitive triplets jumping to  different X |  | **→** |  |  | -0.01 | 0.06 | 14 |  | 0.37 | 0.28 | 9 |

*Significance was tested by dividing the estimates with the standard error, resulting in t values that were approximately normally distributed (Ripley et al., 2020). Convergence statistics: t-ratio all < 0.10, and overall maximum convergence ratio < 0.25. *p ≤ 0.05, **p ≤ 0.01, ***p ≤ 0.001 (two-tailed test).*
